# Short-term mortality after intensive care admission in acute lymphoblastic versus predominantly acute myeloid leukemia: a retrospective cohort study using MIMIC-IV with eICU external replication

**DOI:** 10.3389/fonc.2026.1928507

**Published:** 2026-09-03

**Authors:** Zhe Li, Jianglin Yu

**Affiliations:** 1 Department of Hematology, Guizhou Provincial People’s Hospital, Guiyang, Guizhou, China; 2 The First Clinical College, Zunyi Medical University, Zunyi, Guizhou, China

**Keywords:** acute lymphoblastic leukemia, acute myeloid leukemia, critical care, hematologic neoplasms, intensive care units, mortality, prognosis

## Abstract

**Background:**

Adults with acute leukemia admitted to intensive care have high early mortality. Whether acute lymphoblastic leukemia (ALL) adds prognostic information beyond baseline severity remains clinically important.

**Methods:**

We performed a retrospective MIMIC-IV cohort study of 486 first ICU stays from 372 adults with acute leukemia. Primary outcomes were hospital and ICU mortality. ALL was compared with a predominantly acute myeloid leukemia comparator using complementary confounder-only and severity-adjusted models and ICD-derived remission stratification, with directional replication in an independent eICU cohort.

**Results:**

ALL admissions were younger and less severely ill at ICU entry. Crude hospital mortality was lower in ALL than in the predominantly AML comparator (23.8% vs. 38.6%; P = 0.010). After adjustment for age and comorbidity alone, ALL showed a non-significant reduction in the odds of hospital mortality (adjusted OR, 0.688; 95% CI, 0.385-1.228). Adding first-day Sequential Organ Failure Assessment (SOFA), which partly overlaps with leukemia-related complications, moved this estimate toward the null (adjusted OR, 0.905; 95% CI, 0.485-1.689). ICU-mortality estimates were also non-significant, although the severity-adjusted ICU estimate crossed the null (confounder-only adjusted OR, 0.690; severity-adjusted OR, 1.076). Because first-day SOFA may lie on the subtype-to-outcome pathway, the confounder-only estimate is reported as the total-association estimate (not conditional on the possible mediator), and all confidence intervals remained wide. Model calibration and discrimination were acceptable. In an independent eICU cohort (n=82), the crude and severity-adjusted ALL estimates for hospital mortality (odds ratios 0.667 and 0.445) were likewise non-significant.

**Conclusion:**

In MIMIC-IV, the crude ALL survival advantage narrowed after adjustment and was not statistically significant. In eICU, crude and severity-adjusted estimates were likewise non-significant but moved further from, rather than toward, the null. Wide confidence intervals in both cohorts, the small ALL sample, overlap between first-day SOFA and leukemia-related complications, and unmeasured leukemia-specific factors leave a modest residual subtype effect possible. These are within-ICU associations affected by selection into intensive care; incomplete post-discharge death ascertainment mainly limits longer-horizon interpretation.

## Introduction

Critical illness in acute leukemia remains common and often fatal (1). Adults with acute lymphoblastic leukemia (ALL) or acute myeloid leukemia (AML) may be transferred to the ICU because of sepsis or septic shock (2), respiratory failure, bleeding, metabolic derangement, treatment toxicity, or direct leukemia-related complications (1, 3–5). Outcomes are better than in older historical series, but early mortality is still substantial, and longer survival after ICU admission remains poor in many cohorts (1, 6–9).

In these reports, prognosis after ICU admission tracks organ failure and life-support needs more closely than the leukemia subtype (1, 3, 6, 8–10). Population-based and hematologic-critical-care cohorts show better survival over time, but mortality remains high when severe organ dysfunction, sepsis, or mechanical ventilation is present (6, 8, 9, 11). Danish nationwide data and related ICU-admission studies reach a similar conclusion: ICU admission itself, and survival afterward, depend heavily on age, comorbidity, treatment intensity, and acute support requirements (10–14). Subtype still enters bedside discussions, especially during urgent consultation or triage. Its meaning, however, is difficult to separate from the different case mix and complication profiles seen at ICU entry.

We used MIMIC-IV to ask whether ALL status remains associated with short-term mortality after ICU admission once baseline severity and major case-mix imbalances are considered. Hospital mortality and ICU mortality were prespecified as primary outcomes because they are closest to the ICU episode. To separate subtype from severity, we broke the crude ALL-versus-AML contrast into age, comorbidity, and first-day organ failure. Each model was fitted twice, with and without first-day SOFA, so the conclusion would not turn on one modeling choice. Remission status was read off the ICD codes. Sensitivity analyses covered selection and missing death dates. The MIMIC-IV findings were then checked against an independent eICU cohort. The same analytic sequence (paired severity-adjusted and confounder-only models with covariate-block decomposition) can be reused for other subtype questions in electronic-health-record data. Secondary analyses described acute complication burden and tested whether coded complications added information beyond severity models.

## Materials and methods

### Data source and study design

We performed a retrospective cohort study using MIMIC-IV, a publicly available deidentified electronic health record dataset derived from Beth Israel Deaconess Medical Center (15). The current release contains adult hospital and ICU data from 2008 through 2022 (15). MIMIC-IV is intended to support reproducible research based on routinely collected clinical data (15). MIMIC-IV was approved by the Institutional Review Board at Beth Israel Deaconess Medical Center, which waived the requirement for individual informed consent because the data are fully deidentified. Access required completion of the applicable PhysioNet data use agreement and human-subjects training; no additional participant contact or consent was required for this secondary analysis.

### Cohort definition

Adult admissions were identified from ICD-9 and ICD-10 diagnosis codes recorded during hospitalization. The acute leukemia cohort included admissions coded as acute lymphoblastic leukemia, acute myeloid leukemia (acute monocytic leukemia is part of the AML spectrum and is grouped with AML hereafter), or erythroid leukemia. We scoped the study *a priori* to acute leukemia because the main comparison of interest was ALL versus AML. These diseases share an acute presentation, overlapping ICU organ-failure mechanisms, and similar triage urgency. By contrast, chronic lymphoid neoplasms such as lymphoma or plasma-cell disorders follow different trajectories and fail in different ways in critical care, so they were outside the present question. To avoid overcounting repeated ICU episodes within the same admission, we retained only the first ICU stay of each qualifying hospital admission. Because some patients contributed more than one qualifying admission, we paired the admission-level analysis with a patient-level first-admission sensitivity analysis.

For the primary analysis, admissions were classified as ALL or non-ALL acute leukemia according to whether any ALL code appeared in the admission-level ICD set. Admission-level leukemia labels were built by aggregating distinct matched ICD categories. For descriptive subgroup summaries, we further collapsed the observed categories into ALL, AML, and AML-monocytic-inclusive. The AML-monocytic-inclusive subgroup contained admissions labeled AML-monocytic alone and the single admission labeled AML and AML-monocytic. No erythroid leukemia admission met the final ICU cohort definition. Throughout, “non-ALL” denotes all acute leukemia admissions that are not ALL, and “AML” denotes AML without a monocytic code. The 402 non-ALL ICU admissions comprised 395 AML, 6 AML-monocytic-only, and 1 admission co-coded AML and AML-monocytic; this last group was displayed descriptively as AML-monocytic-inclusive (n=7).

ICU conditioning creates a selection problem: the patients who reach the ICU may not represent the underlying leukemia subgroups in the same way. For that reason, ICU admission rates for ALL and AML were compared across the full adult acute leukemia admission pool. We also ran a marginal subtype-balance check using subtype-specific ICU admission probabilities from the full ALL/AML-coded pool. This check reweighted the overall ALL-to-AML mix only. It did not model bedside triage or within-subtype ICU selection and was interpreted accordingly.

### Variables

Baseline variables covered demographics (age, sex, race, insurance, marital status), admission features (admission type, admission location, and first ICU care unit), comorbidity and severity, selected first-day laboratory measurements, and early organ support during the first 24 h after ICU admission. Severity variables included Charlson comorbidity index, first-day SOFA score, SAPS II, lowest first-day Glasgow Coma Scale score, and first-day urine output. SOFA and SAPS II were included because both are established severity indices in critical care research (16, 17). Each was calculated over the first ICU day using the standard MIMIC-IV derived concepts. We used first-day SOFA as a near-presentation severity variable, while recognizing that some components may overlap with downstream leukemia-related complications. Acute complications were identified from all ICD-9 and ICD-10 diagnosis codes recorded during the qualifying admission and included sepsis or septic shock, respiratory failure, pneumonia, acute kidney injury, coagulopathy, bleeding, or anemia, neutropenia, and tumor lysis syndrome. Discharge disposition and survival endpoints were not used as predictors.

### Outcomes

The primary outcomes were hospital mortality and ICU mortality. Secondary outcomes were fixed-time all-cause mortality at 90 and 365 days from ICU admission. Post-discharge death status came from in-hospital death documentation and the patient-level date-of-death (DOD) field in MIMIC-IV. When no death date was recorded, the patient was not classified as dead at that landmark. Follow-up completeness was reported overall and among hospital survivors; for patients with a post-discharge death date, we described time from discharge to death. Because missing post-discharge DOD could bias fixed-time mortality estimates, we calculated survivor-only extreme-case ranges for 365-day mortality by assuming that all hospital survivors without recorded DOD were either alive or dead by 365 days. We also analyzed 365-day all-cause survival from ICU admission using Kaplan–Meier estimation, log-rank testing, and Cox regression. Patients without a death record were censored at 365 days. These Cox analyses were considered supportive because individual post-discharge censoring dates were unavailable.

### Statistical analysis

We structured and reported the study following STROBE and RECORD guidance (18, 19). Continuous variables are reported as median with interquartile range, and categorical variables as counts with percentages. Baseline characteristics in **Table 1** are descriptive. Crude outcome comparisons used Pearson chi-square tests, with Fisher exact test reserved for sparse tables. Missingness was reported for manuscript-level variables as the number and percentage of missing observations. An external replication in the eICU collaborative research database repeated the primary ALL-versus-predominantly-AML comparison with hospital and unit mortality as outcomes and APACHE score as the severity adjuster (**Supplementary File 16**).

**Table 1:** Baseline characteristics of adult acute leukemia ICU admissions by ALL status.

Variable	Available n	Missing n (%)	Overall	ALL	Non-ALL
Age, years	486	0 (0.0%)	63.0 [52.0, 72.0]	51.0 [34.0, 61.2]	65.0 [54.0, 74.0]
Male sex	486	0 (0.0%)	293 (60.3%)	40 (47.6%)	253 (62.9%)
Race	486	–			
White			358 (73.7%)	53 (63.1%)	305 (75.9%)
Black			20 (4.1%)	7 (8.3%)	13 (3.2%)
Asian			21 (4.3%)	6 (7.1%)	15 (3.7%)
Hispanic			18 (3.7%)	7 (8.3%)	11 (2.7%)
Other/unknown			69 (14.2%)	11 (13.1%)	58 (14.4%)
Insurance	486	–			
Medicare			260 (53.5%)	30 (35.7%)	230 (57.2%)
Private			151 (31.1%)	36 (42.9%)	115 (28.6%)
Medicaid			56 (11.5%)	14 (16.7%)	42 (10.4%)
Other/unknown			19 (3.9%)	4 (4.8%)	15 (3.7%)
Marital status	486	–			
MARRIED			261 (53.7%)	40 (47.6%)	221 (55.0%)
SINGLE			138 (28.4%)	32 (38.1%)	106 (26.4%)
Other/unknown			87 (17.9%)	12 (14.3%)	75 (18.7%)
Admission type	486	–			
Emergency			325 (66.9%)	63 (75.0%)	262 (65.2%)
Observation			92 (18.9%)	15 (17.9%)	77 (19.2%)
Urgent			58 (11.9%)	6 (7.1%)	52 (12.9%)
Elective/other			11 (2.3%)	0 (0.0%)	11 (2.7%)
Admission location	486	–			
Emergency room			179 (36.8%)	33 (39.3%)	146 (36.3%)
Transfer from hospital			96 (19.8%)	15 (17.9%)	81 (20.1%)
Physician referral			112 (23.0%)	15 (17.9%)	97 (24.1%)
Other			99 (20.4%)	21 (25.0%)	78 (19.4%)
First ICU care unit	486	–			
MICU/SICU			385 (79.2%)	58 (69.0%)	327 (81.3%)
MICU			34 (7.0%)	6 (7.1%)	28 (7.0%)
CCU			19 (3.9%)	7 (8.3%)	12 (3.0%)
Other			48 (9.9%)	13 (15.5%)	35 (8.7%)
Charlson comorbidity index	486	0 (0.0%)	6.0 [4.0, 7.0]	5.0 [3.0, 6.0]	6.0 [4.0, 7.0]
First-day SOFA	486	0 (0.0%)	6.0 [4.0, 8.0]	4.5 [3.0, 7.0]	6.0 [4.0, 8.0]
SAPS II	486	0 (0.0%)	44.0 [36.0, 55.0]	38.0 [30.0, 47.0]	46.0 [37.0, 55.0]
First-day GCS minimum	481	5 (1.0%)	15.0 [14.0, 15.0]	15.0 [14.0, 15.0]	15.0 [14.0, 15.0]
First-day urine output, mL	463	23 (4.7%)	1,630.0 [917.0, 2640.0]	1,957.5 [1164.0, 2936.2]	1,550.0 [872.5, 2547.5]
Hemoglobin minimum, g/dL	481	5 (1.0%)	7.7 [6.8, 8.9]	8.6 [7.5, 9.9]	7.6 [6.7, 8.7]
WBC maximum, K/µL	477	9 (1.9%)	6.5 [1.2, 18.2]	6.2 [1.7, 16.3]	6.5 [1.1, 18.6]
Platelet minimum, K/µL	481	5 (1.0%)	36.0 [16.0, 91.0]	64.0 [32.5, 188.0]	31.0 [15.0, 81.0]
Creatinine maximum, mg/dL	480	6 (1.2%)	1.1 [0.8, 1.7]	0.9 [0.7, 1.4]	1.2 [0.8, 1.7]
BUN maximum, mg/dL	479	7 (1.4%)	24.0 [16.0, 37.0]	20.0 [13.0, 27.8]	25.0 [17.0, 39.0]
Invasive ventilation within 24 h	486	0 (0.0%)	103 (21.2%)	17 (20.2%)	86 (21.4%)
Any vasoactive agent within 24 h	486	0 (0.0%)	100 (20.6%)	14 (16.7%)	86 (21.4%)
Dialysis within 24 h	486	0 (0.0%)	29 (6.0%)	2 (2.4%)	27 (6.7%)
ICU length of stay, days	485	1 (0.2%)	1.91 [1.05, 4.00]	1.91 [0.90, 3.30]	1.91 [1.10, 4.20]
Hospital length of stay, days	486	0 (0.0%)	18.9 [7.8, 38.8]	20.5 [8.2, 36.4]	18.9 [7.5, 39.1]
ICU death	486	0 (0.0%)	82 (16.9%)	9 (10.7%)	73 (18.2%)
Hospital death	486	0 (0.0%)	175 (36.0%)	20 (23.8%)	155 (38.6%)

Continuous variables are presented as median [IQR], and categorical variables as n (%). The cohort includes only the first ICU stay of each qualifying hospital admission. MICU/SICU and MICU are distinct original MIMIC-IV care unit labels. For race, insurance, and marital status, the “Other/unknown” category combines patients whose value was not recorded with those selecting an alternative option, so these variables show no separate missing count. Available n denotes the number of non-missing observations for the corresponding variable. Raw missingness counts for these variables before absorption into the Other/unknown category are provided in **Supplementary File 8** (Variable Missingness).

Because baseline differences between ALL and non-ALL admissions were pronounced, we used multivariable analyses to estimate the adjusted association between ALL status and mortality. Ordinary maximum-likelihood logistic regression was used for all prespecified fixed-time models. All models converged, with no evidence of numerical separation. Regression analyses used complete-case data for the variables included in each model, and P values were derived from Wald statistics. The primary-model covariates (ALL status, age, sex, Charlson comorbidity index, and first-day SOFA) were complete for all 486 admissions, so the primary analysis required no imputation; sensitivity models that added laboratory covariates retained at least 476 of 486 admissions (**Table 2**). Complete-case analyses were supported by a missingness comparison and a multiple-imputation sensitivity analysis (**Supplementary File 15**): the 10 admissions missing white-cell or platelet counts did not differ materially from complete cases, and multiple imputation gave an essentially identical ALL estimate for the extended hematologic model. Events-per-variable were 35 for hospital mortality and 16 for ICU mortality. An unadjusted two-proportion power calculation indicated 80% power to detect large hospital-mortality effects (ALL mortality ≤23.1% or ≥55.3%, i.e., OR ≤0.48 or ≥1.97) but, for ICU mortality, only OR ≤0.35 or ≥2.19 (observed *post-hoc* power 43%); the adjusted analysis has lower effective power, so smaller adjusted effects cannot be excluded.

**Table 2:** Adjusted associations between ALL status and short-term mortality in adult acute leukemia ICU admissions.

Analysis	Outcome	N	Events	Adjusted OR for ALL	95% CI	P value	C-statistic	Brier
Severity-adjusted primary	Hospital mortality	486	175	0.905	0.485 to 1.689	0.753	0.769	0.185
Severity-adjusted primary	ICU mortality	486	82	1.076	0.455 to 2.540	0.868	0.813	0.109
SAPS II substitution	Hospital mortality	486	175	0.790	0.425 to 1.466	0.455	0.761	0.189
SAPS II substitution	ICU mortality	486	82	0.892	0.382 to 2.080	0.791	0.818	0.112
Ventilation-adjusted sensitivity	Hospital mortality	486	175	0.937	0.500 to 1.758	0.840	0.770	0.185
Ventilation-adjusted sensitivity	ICU mortality	486	82	0.994	0.418 to 2.363	0.990	0.824	0.108
Extended hematologic	Hospital mortality	476	170	1.112	0.572 to 2.163	0.755	0.786	0.178
Extended hematologic	ICU mortality	476	81	0.978	0.393 to 2.437	0.963	0.820	0.109
Sensitivity vasoactive	Hospital mortality	486	175	0.942	0.501 to 1.771	0.853	0.772	0.184
Sensitivity vasoactive	ICU mortality	486	82	0.990	0.411 to 2.383	0.982	0.827	0.107
Patient-level first admission primary model	Hospital mortality	372	137	0.858	0.409 to 1.800	0.685	0.787	0.179
Patient-level first admission primary model	ICU mortality	372	69	1.221	0.481 to 3.098	0.674	0.820	0.114
ALL versus AML only primary model	Hospital mortality	479	174	0.914	0.486 to 1.716	0.779	0.771	0.185
ALL versus AML only primary model	ICU mortality	479	81	0.978	0.410 to 2.332	0.960	0.823	0.108

The primary model included ALL status, age, sex, Charlson comorbidity index, and first-day SOFA score. Short-term sensitivity analyses included ventilation-adjusted, patient-level first-admission, ALL-versus-AML-only, extended hematologic, vasoactive-agent, and SAPS II-substitution models. Follow-up (90- and 365-day) and Kaplan–Meier/Cox mortality analyses are reported in the Supplementary Material (**Supplementary Figure 3**) because post-discharge date-of-death ascertainment was incomplete; extreme-case bounds and tipping-point analyses are also provided there. C-statistics are apparent values except for the primary models, whose optimism-corrected values were 0.760 for hospital mortality and 0.802 for ICU mortality. P values for sensitivity models are not adjusted for multiplicity.

Two adjusted estimates were reported for the ALL-mortality association. The confounder-only model included ALL status, age, sex, and Charlson comorbidity index, but not first-day SOFA; the severity-adjusted primary model then added first-day SOFA. The severity-adjusted model is the primary model; the confounder-only model is reported alongside as the total-effect estimate that is not conditional on near-presentation severity. All covariates were available at or near ICU presentation. Because some SOFA components may lie on a subtype-to-outcome pathway, the two estimates address different questions: the confounder-only estimate reflects the overall association, and the severity-adjusted estimate the direct association conditional on near-presentation severity. We drew an inference from their agreement, not from either alone, so the conclusion does not depend on whether the possible mediator is included. The same covariate framework was applied to 90- and 365-day fixed-time mortality from ICU admission. For the time-to-event extension, 365-day Cox models used the same baseline covariate set. A Schoenfeld residual test suggested possible non-proportionality for continuous SOFA in the ICU-origin Cox model, so we additionally fit a sensitivity Cox model stratified by SOFA tertile.

To address calendar time, we reconstructed approximate real-world year using the MIMIC-IV privacy-preserving date-offset structure: the midpoint of the reported anchor-year interval plus the difference between the admission year and the patient-specific anchor year. No additional integer rounding was applied before era assignment. This midpoint reconstruction can map a small number of admissions near an interval boundary slightly outside the 2008–2022 data-collection window. Those admissions (n=4) were excluded from the era-banded model only and retained in all other analyses. Admissions were then grouped into prespecified eras (2008-2013, 2014-2017, and 2018-2022), and a COVID-period sensitivity window was defined as 2020-2022. Further sensitivity analyses examined whether the ALL estimate changed meaningfully after adding very early respiratory support, using invasive ventilation within the first 24 h as an example.

Additional sensitivity analyses used subject-level cluster-robust standard errors to account for repeated admissions from the same patient. They also included a patient-level first-admission model, an ALL-versus-AML-only model that excluded the seven non-AML, non-ALL admissions, and a SAPS II substitution model that replaced SOFA with SAPS II. The extended hematologic model added white blood cell count maximum and platelet count minimum, with limited case loss. A separate sensitivity model added vasoactive-agent exposure while leaving the remaining covariates unchanged. The marginal subtype-balance check used simple stabilized weights derived only from subtype-specific ICU admission probabilities in the full adult ALL/AML-coded admission pool. Because it could not address within-subtype ICU triage selection, we interpreted it only as a marginal-balance sensitivity check.

Exploratory complication-augmented models first added admission-level sepsis alone and then sepsis, respiratory failure, and acute kidney injury to the ventilation-adjusted model to test whether coded acute complications carried prognostic information beyond severity. Because these complication flags came from admission-wide ICD coding rather than from time-anchored ICU baseline ascertainment, we interpreted them as exploratory rather than primary prognostic models. Model discrimination was summarized with the C-statistic, calibration with the Hosmer–Lemeshow test (unstable when expected cell counts are small, as for ICU mortality with 82 events), and with 10-fold cross-validated calibration curves, and overall predictive performance with the Brier score. These metrics are reported as descriptive summaries of association strength and model fit rather than as a validated clinical prediction model. For the primary model, we additionally report the optimism-corrected C-statistic from a Harrell bootstrap with 1,000 admission-level resamples (resampling admissions rather than patients; the patient-level cluster structure from repeated admissions is addressed separately by the cluster-robust sensitivity analysis). Logistic-model AUCs and Cox-model concordance statistics were reported separately and were not treated as directly interchangeable. Collinearity was assessed with variance inflation factors (VIFs).

We quantified attenuation with sequential logistic models: ALL alone; ALL plus age, sex, and Charlson comorbidity index; and the primary model after adding first-day SOFA. Each block’s share was calculated from its contribution to the total change in the ALL log-odds between the crude and full models. Because this calculation depends on covariate order, we also report each block’s incremental value when entered last, using likelihood-ratio tests and the change in C-statistic (**Table 3**; **Supplementary Material**). Sensitivity to unmeasured confounding was summarized with E-values for the primary estimates and confidence limits. An E-value is the minimum risk-ratio strength of association that an unmeasured confounder would need with both subtype and mortality to move the estimate to the null. E-values do not formally address collider or selection bias from conditioning on ICU admission, which is handled as a separate limitation.

**Table 3:** Covariate-block decomposition of the ALL-mortality association (n=486, complete-case).

Outcome	Model	ALL aOR (95% CI)	P	C-stat	Attenuation share
Hospital mortality	ALL only (crude)	0.498 (0.290-0.855)	0.012	0.546	–
Hospital mortality	+ Age/sex/Charlson	0.688 (0.385-1.228)	0.206	0.654	54%
Hospital mortality	+ SOFA (primary model)	0.905 (0.485-1.689)	0.753	0.769	46%
ICU mortality	ALL only (crude)	0.541 (0.259-1.130)	0.102	0.538	–
ICU mortality	+ Age/sex/Charlson	0.690 (0.320-1.492)	0.346	0.611	36%
ICU mortality	+ SOFA (primary model)	1.076 (0.455-2.540)	0.868	0.813	64%

Sequential cumulative logistic models show the ALL odds ratio and the C-statistic at each step; the attenuation share is each block’s contribution to the total change in the ALL log-odds between the crude and full (primary) models. C-statistics are Mann–Whitney AUCs of the fitted model. ALL, acute lymphoblastic leukemia; SOFA, Sequential Organ Failure Assessment. Incremental-value estimates (each block entered last) and standalone single-block C-statistics are provided in the **Supplementary Material**. P values are not adjusted for multiplicity.

## Results

### Cohort characteristics

**Figure 1:**
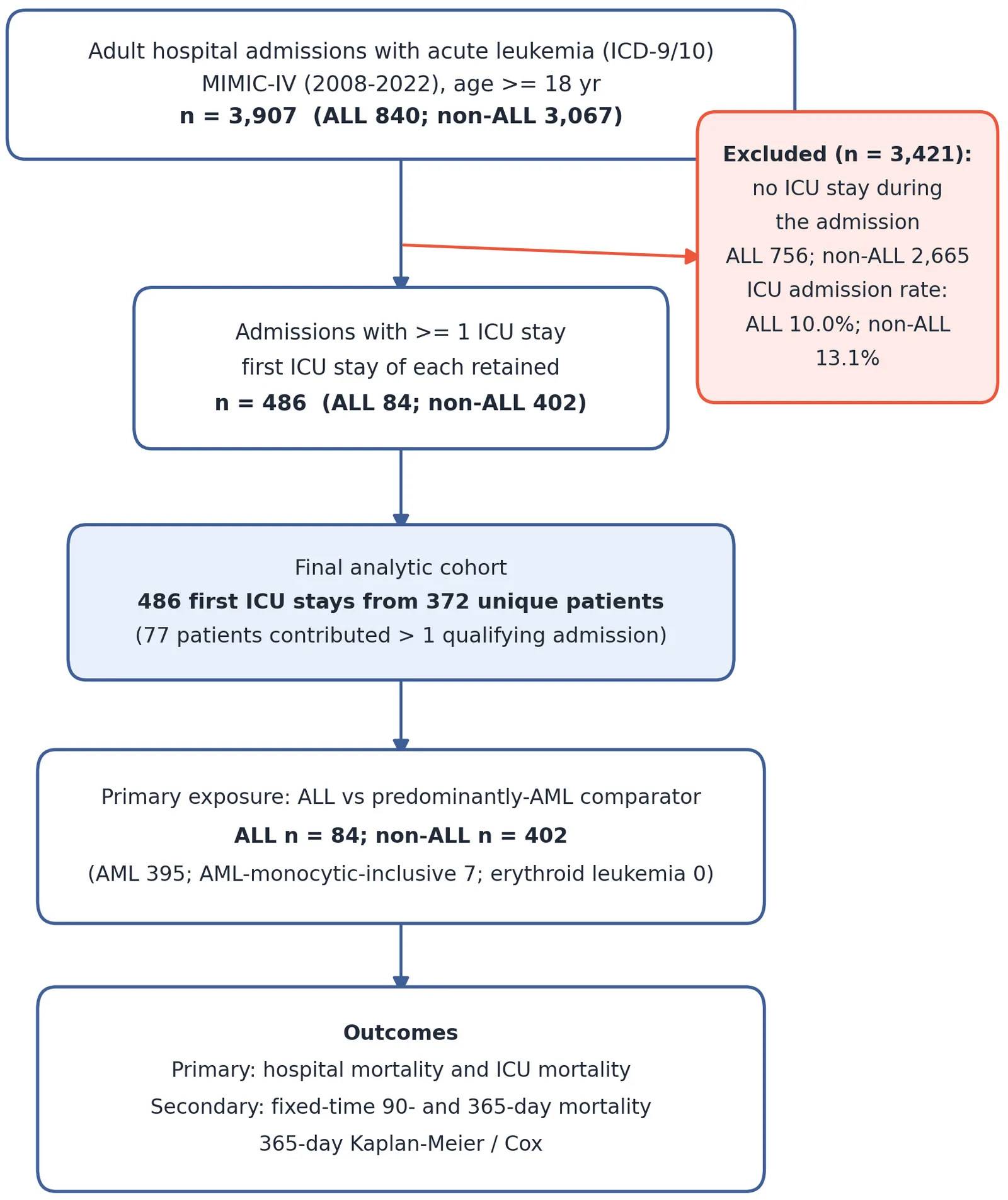
Cohort flow diagram showing derivation of the adult acute leukemia ICU cohort from MIMIC-IV. The analytic sample was restricted first to adult acute leukemia admissions identified by ICD-9/10 coding and then to the first ICU stay of each qualifying admission.

The final cohort comprised 486 first ICU stays from hospital admissions with acute leukemia: 84 ALL admissions and 402 non-ALL admissions. These stays came from 372 unique patients, 77 of whom contributed more than one qualifying admission. Cohort derivation is shown in **Figure 1**.

### Cohort case mix

The cohort covered a broad acuity range. A first-day SOFA score of at least 6 was present in 52.1% of admissions, yet only 21.2% received invasive ventilation, 20.6% received any vasoactive agent, and 6.0% received renal replacement therapy within the first 24 h; 66.7% received none of these invasive supports. The median ICU length of stay was 1.91 days (IQR 1.05-4.00). Acute complications were nevertheless common at the admission level, including acute kidney injury (51.0%), coagulopathy, bleeding, or anemia (49.2%), respiratory failure (49.0%), sepsis or septic shock (41.6%), neutropenia (39.7%), and pneumonia (37.2%). These complication rates were derived from admission-wide ICD coding and describe admission burden rather than ICU-presentation state, so they are not directly comparable with the time-anchored 24-h organ-support figures above.

Within the non-ALL cohort, 395 admissions were AML (AML without a monocytic code), 6 were labeled AML-monocytic, and 1 carried both AML and AML-monocytic labels.

Before adjustment, the ALL subgroup differed markedly from the predominantly AML comparator at ICU presentation (**Table 1**). Patients with ALL were younger than those in the predominantly AML comparator (median age 51.0 years [IQR, 34.0-61.2] vs. 65.0 years [IQR, 54.0-74.0]). The Charlson comorbidity index was lower in ALL (5.0 [3.0-6.0] vs. 6.0 [4.0-7.0]), as were first-day SOFA scores (4.5 [3.0-7.0] vs. 6.0 [4.0-8.0]). SAPS II showed the same direction (38.0 [30.0-47.0] vs. 46.0 [37.0-55.0]). These baseline differences motivated the adjusted analyses rather than serving as independent hypothesis tests.

Crude hospital mortality was lower in ALL than in the predominantly AML comparator (20/84 [23.8%] vs. 155/402 [38.6%]; P = 0.010). Crude ICU mortality, however, did not differ significantly (9/84 [10.7%] vs. 73/402 [18.2%]; P = 0.098). Crude follow-up mortality from ICU admission was also lower in ALL at both 90 days (28/84 [33.3%] vs. 208/402 [51.7%]; P = 0.002) and 365 days (42/84 [50.0%] vs. 273/402 [67.9%]; P = 0.002).

### Adjusted analyses

**Figure 2:**
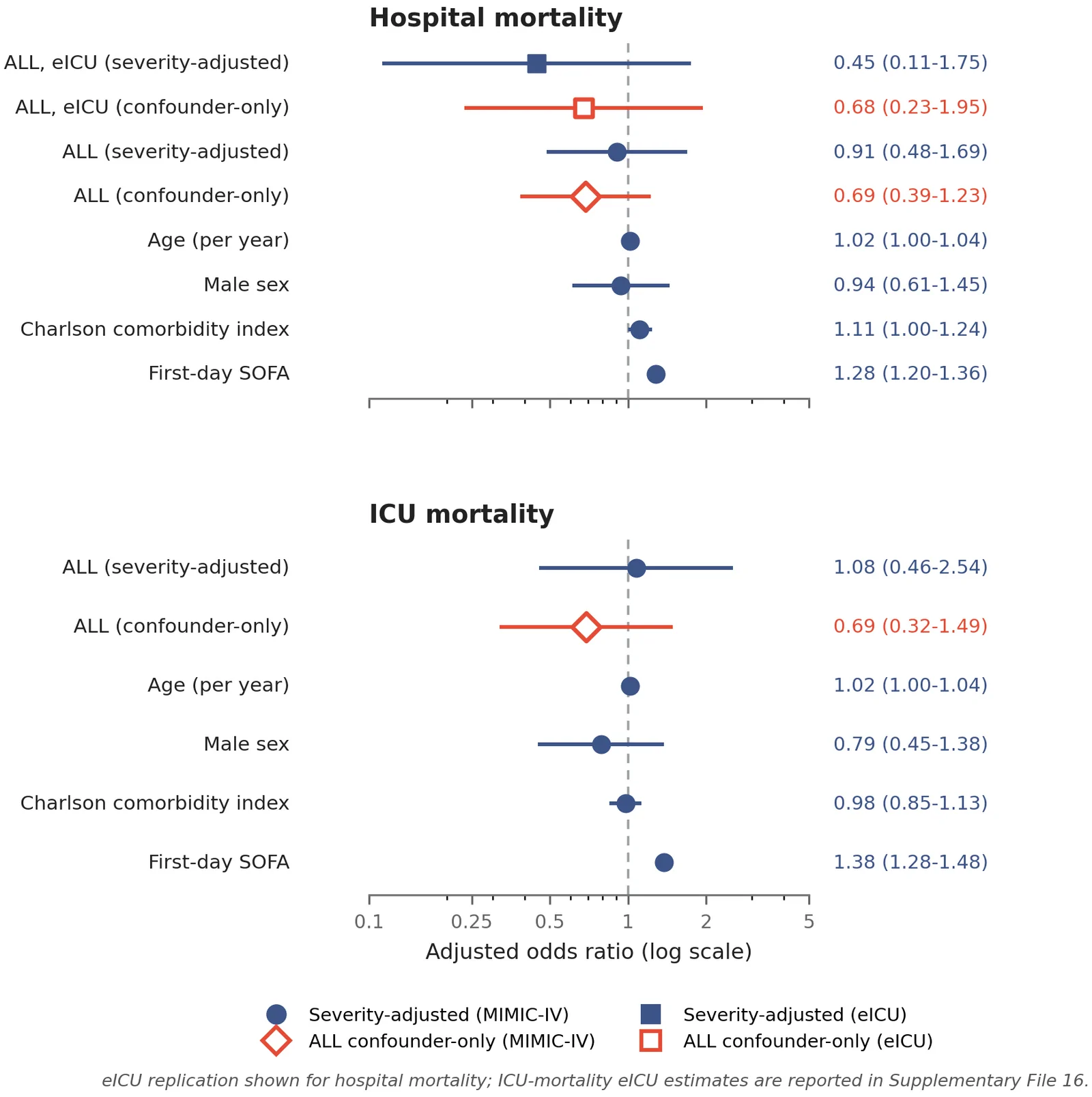
Forest plot of odds ratios for hospital mortality and ICU mortality. In MIMIC-IV, filled circles show the severity-adjusted primary model, which included ALL status, age, sex, Charlson comorbidity index, and first-day SOFA score; open diamonds show the confounder-only ALL estimate fitted without first-day SOFA. In the hospital-mortality panel, filled and open squares show the corresponding eICU severity-adjusted and confounder-only estimates, respectively. eICU unit-mortality estimates are reported in **Supplementary File 16**. Odds ratios are shown on a logarithmic scale with 95% confidence intervals.

All prespecified logistic models converged successfully. Collinearity was minimal; all evaluated variance inflation factors were below 2.0 (maximum 1.70 for age), indicating no concerning multicollinearity. In the severity-adjusted primary model (ALL versus the predominantly AML comparator), ALL status was not independently associated with hospital mortality (adjusted OR, 0.905; 95% CI, 0.485-1.689; P = 0.753). The ICU-mortality estimate was also non-significant (adjusted OR, 1.076; 95% CI, 0.455-2.540; P = 0.868). The intervals were wide, so a modest subtype effect could not be excluded. First-day SOFA remained independently associated with both short-term endpoints, and older age was associated with hospital mortality. These primary adjusted associations are displayed in **Figure 2**. Apparent in-sample fit was acceptable. C-statistics were 0.769 for hospital mortality and 0.813 for ICU mortality (optimism-corrected to 0.760 and 0.802 after 1,000-resample Harrell bootstrap), with Brier scores of 0.185 and 0.109 and Hosmer–Lemeshow (HL) P values of 0.171 and 0.356, respectively. Tenfold cross-validated calibration slopes stayed close to 1.0 (**Supplementary Figure 1**).

The confounder-only model, which omitted SOFA, gave an adjusted OR for ALL of 0.688 (95% CI, 0.385-1.228; P = 0.206) for hospital mortality and 0.690 (95% CI, 0.320-1.492; P = 0.346) for ICU mortality. Reintroducing first-day SOFA shifted both estimates toward 1.0, to 0.905 and 1.076, respectively, with the ICU estimate crossing the null.

Calendar-time sensitivity analyses supported the primary result. Era-banded crude hospital mortality remained lower in ALL than in the predominantly AML comparator across 2008-2013, 2014-2017, and 2018-2022. Because privacy-shifted year reconstruction mapped four admissions slightly beyond the prespecified 2008–2022 window, the era-banded model included 482 admissions. After era indicators were added to the primary model, the adjusted OR for ALL was 0.932 (95% CI, 0.496-1.750; P = 0.826) for hospital mortality and 1.065 (95% CI, 0.447-2.538; P = 0.886) for ICU mortality. The ALL × late-era (2018-2022) interaction was not significant for hospital mortality (OR, 1.524; 95% CI, 0.404-5.751; P = 0.534). For ICU mortality, the interaction estimate was larger but imprecise (OR, 4.333; 95% CI, 0.777-24.144; P = 0.094), reflecting the small number of ICU deaths. The COVID-period window (2020-2022) contributed 84 admissions, including 17 ALL and 67 non-ALL. Era-stratified crude counts and the full era-adjusted, interaction, and COVID-period model estimates are provided in the **Supplementary Material**.

Other short-term sensitivity analyses remained non-significant, including the ventilation-adjusted, patient-level first-admission, ALL-versus-AML-only, cluster-robust, SAPS II substitution, extended hematologic, and vasoactive-agent models, and a remission-adjusted model (**Supplementary File 14**). A marginal subtype-balance check in the ALL-versus-AML-coded pool (n=480 of 486, excluding the six admissions with no ALL or AML code) gave a consistent non-significant ALL estimate (Supplementary Material). Remission status was derived from the 6th-digit ICD code specificity. Active disease was strongly associated with hospital mortality compared with in-remission status (adjusted OR 4.25, 95% CI 1.94-9.31; P<0.001), but not with ICU mortality. Adding remission status did not materially change the ALL estimate (**Supplementary File 14**). Restricting the comparator to AML-only admissions produced a nearly identical hospital-mortality estimate for ALL (adjusted OR, 0.914; 95% CI, 0.486-1.716; P = 0.779), suggesting that the seven non-AML, non-ALL admissions had little impact on the primary comparison.

ALL also did not add incremental prognostic information beyond a base severity model: adding ALL status failed to improve discrimination or likelihood-based fit for either hospital mortality (likelihood-ratio chi-square 0.099; P = 0.752) or ICU mortality (0.027; P = 0.868).

A covariate-block decomposition quantified how the crude ALL contrast was absorbed (**Table 3**). For hospital mortality, adding age, sex, and Charlson comorbidity index moved the ALL estimate from 0.498 (crude) to 0.688, accounting for 54% of the total log-odds attenuation, and adding first-day SOFA moved it to 0.905 (the remaining 46%). For ICU mortality, SOFA accounted for a larger share (64%) and demographics for 36%. SOFA carried the strongest single signal. Entered last, it improved the C-statistic by 0.116 for hospital mortality and 0.202 for ICU mortality, with likelihood ratio P<0.001 for both. ALL status, entered last, changed the C-statistic by less than 0.001 and was not significant (P = 0.752 hospital; P = 0.868 ICU). Because the C-statistic is relatively insensitive to a single binary predictor, this small change should not be read as proof of no subtype effect; the likelihood-ratio test is the more appropriate incremental test. Because first-day SOFA partly lies on a subtype-to-outcome pathway (**Supplementary Figure 2**), the near-zero incremental value of ALL beyond SOFA is consistent with overadjustment and does not exclude a clinically meaningful subtype effect.

Exploratory complication-augmented models are reported in the **Supplementary Material**. The admission-wide ICD complication flags may capture events on the pathway to death rather than baseline prognostic markers. The complication-augmented hospital-mortality model also showed significant miscalibration (Hosmer–Lemeshow P = 0.016). For these reasons, the results are not treated as independent prognostic information and do not alter the primary short-term conclusion.

Longer-horizon analyses followed the same broad pattern, but they are secondary because post-discharge ascertainment through the DOD field was incomplete. DOD was recorded for 375/486 admissions (77.2%) and for 200/311 hospital survivors (64.3%); survivors without DOD were treated as alive at the fixed landmark. Among hospital survivors with a recorded post-discharge death, median time from discharge to death was 170.3 days (IQR 40.3-439.6). The uncertainty was large. Under extreme-case assumptions, 365-day mortality among hospital survivors ranged from 45.0% (if all 111 survivors without a recorded death date were alive) to 80.7% (if all had died). Cohort-level 365-day mortality ranged from 64.8% to 87.7%. ALL status was not independently associated with 90-day mortality (adjusted OR 0.947, 95% CI 0.534-1.681) or 365-day mortality (0.957, 0.547-1.675). Tipping-point analyses showed that the 365-day estimate remained non-significant under symmetric or extreme imputation of the missing death dates (**Supplementary Material**). Baseline-adjusted Cox models were likewise null, including a SOFA-stratified sensitivity model (**Supplementary Figure 3**).

Across all adult acute leukemia admissions, ICU admission differed by subtype: 10.0% of ALL-coded admissions (84/840) versus 13.1% of non-ALL admissions (402/3,067) were admitted to the ICU (rate ratio 0.76; P = 0.018). Tipping-point, complication-augmented, and other sensitivity analyses are presented in the **Supplementary Material**. Using the standard common-outcome approach implemented in the EValue package, the point-estimate E-values were 1.28 for hospital mortality and 1.23 for ICU mortality; because both confidence intervals already crossed the null, the corresponding CI E-values were 1.00 (**Supplementary Material**). E-values address unmeasured confounding and do not formally bound the selection bias from conditioning on ICU admission, which remains a residual limitation.

### Descriptive subgroup analysis

The observed ICU subgroups were ALL (n=84), AML (n=395), and AML-monocytic-inclusive (n=7). No erythroid leukemia admission met the final ICU cohort definition. Crude hospital mortality was 23.8% in ALL, 39.0% in AML, and 1/7 (14.3%) in AML-monocytic-inclusive admissions. Given the very small AML-monocytic-inclusive subgroup, these results are descriptive only.

### External replication in eICU

To assess whether the short-term pattern generalized beyond MIMIC-IV, we repeated the ALL-versus-predominantly-AML comparison in an independent eICU cohort (**Supplementary File 16**). Adults (18 years or older) with a first ICU stay and acute leukemia, identified from free-text diagnosis and APACHE admission-diagnosis fields, comprised 82 stays (52 ALL and 30 non-ALL, of whom 25 were AML). Because eICU encodes severity as APACHE rather than SOFA and has no Charlson comorbidity index, the severity-adjusted model used APACHE score in place of SOFA and the confounder-only model used age and sex only; these specifications therefore differ from the MIMIC-IV primary models. As in MIMIC-IV, ALL admissions were younger (median age 58 vs. 63 years) and had lower crude hospital mortality (13/52 [25.0%] vs. 10/30 [33.3%]). The crude hospital-mortality odds ratio for ALL was 0.667 (95% CI 0.249-1.785); the confounder-only estimate was 0.675 (0.234-1.949); the severity-adjusted estimate was 0.445 (0.113-1.752). Unit-mortality estimates were similarly non-significant (severity-adjusted OR 0.388, 95% CI 0.072-2.099). In the cleaner ALL-versus-AML-only subcomparison, the severity-adjusted hospital-mortality OR was 0.470 (95% CI 0.113-1.960). All confidence intervals were wide and crossed the null. The eICU point estimates lie on the same (protective) side of the null as the MIMIC-IV crude and confounder-only estimates, and the two cohorts’ 95% confidence intervals overlap broadly. One difference should be stated plainly: in MIMIC-IV, severity adjustment moved the ALL estimate toward the null (0.498 to 0.905), whereas in eICU, it moved it further from the null (0.667 to 0.445); the attenuation pattern central to the MIMIC-IV analysis was therefore not reproduced in eICU. Because the eICU cohort is small (n=70 in the severity-adjusted model, 20 hospital events), this directional difference may reflect noise rather than a genuine difference in mechanism. Both cohorts support the same, narrow inference: a crude ALL advantage that does not reach statistical significance after adjustment, and too imprecise to confirm or exclude a modest subtype effect. Hospital-discharge mortality was complete for all 82 eICU stays, so this comparison is not affected by the post-discharge date-of-death ascertainment gap that limits the MIMIC-IV longer-horizon analyses.

## Discussion

In this MIMIC-IV cohort of adults with acute leukemia requiring ICU care, ALL was linked to lower crude hospital mortality. That apparent advantage did not persist after adjustment for age, comorbidity burden, and baseline severity. At the crude level, the same directional pattern appeared in an independent eICU cohort (n=82): ALL admissions were younger and had lower hospital mortality. Its adjusted estimates were also non-significant with wide confidence intervals (**Supplementary File 16**). Unlike in MIMIC-IV, severity adjustment did not move the eICU estimate toward the null, a divergence the small eICU sample cannot resolve. The strongest inference concerns early hospital and ICU mortality within ICU-selected cohorts, not longer-horizon survival. Across the confounder-only and severity-adjusted primary models, the ALL estimate stayed close to the null and precision was limited. Baseline severity showed the clearest measured association, consistent with earlier studies (1, 3, 6, 9, 10). What this study adds is not a new clinical answer: the qualitative conclusion is consistent with recent meta-analytic evidence (1). The contribution is a two-database directional replication, in which the crude ALL advantage did not reach statistical significance after adjustment in either MIMIC-IV or eICU, and severity and remission carried the clearest measured short-term signal.

The paired SOFA models clarify the adjustment pattern. Without first-day SOFA, the adjusted ALL estimate remained below 1.0 for both hospital and ICU mortality. After SOFA was added, both estimates moved toward the null. Because SOFA components may overlap with downstream leukemia-related complications, this movement is interpreted as severity adjustment rather than causal mediation. The confounder-only estimate (hospital-mortality adjusted OR 0.688, 95% CI 0.385-1.228) was also non-significant. The covariate-block decomposition (**Table 3**) showed that attenuation was shared between demographic/comorbidity factors and first-day SOFA. SOFA was still the strongest single discriminator and added the largest incremental value when entered last. Consistent with residual overadjustment in the SOFA model, substituting SAPS II moved the hospital-mortality estimate for ALL toward protection (adjusted OR 0.790, 95% CI 0.425-1.466, versus 0.905 with SOFA). SAPS II weights physiologic inputs differently from SOFA and may therefore capture less of the acute leukemia-related derangement. Both estimates remained non-significant, so the discrepancy is at least as compatible with residual overadjustment as with absence of a subtype effect.

Remission status reinforced this picture. Active disease versus in-remission was extracted coarsely from the 6th-digit ICD specificity and was the largest leukemia-specific statistical signal in the cohort (adjusted OR 4.25, 95% CI 1.94-9.31; P<0.001). The higher in-remission rate among ALL admissions (22.6% vs. 16.2%) is consistent with the direction of their lower crude mortality, although this subgroup difference was itself not statistically significant (P = 0.16). The ALL estimate changed little after remission was added. These findings carry two caveats: the remission variable derives from administrative coding of uncertain accuracy, and any non-null leukemia-specific variable will outweigh a subtype label whose adjusted effect is near the null.

The case-mix results add context. Two-thirds of admissions did not receive invasive ventilation, vasoactive agents, or dialysis within the first 24 h, yet admission-level coding showed frequent acute kidney injury, bleeding, coagulopathy, or anemia, respiratory failure, sepsis, and neutropenia. The cohort therefore spans several clinical scenarios: invasive critical care, short-stay monitoring, non-invasive support, and transfusion-intensive management of leukemia-related complications. These differences were not fully captured by leukemia subtype alone. The results should therefore be generalized to mixed-acuity ICU leukemia cohorts rather than to a population composed only of patients receiving invasive organ support.

Calendar time did not change the interpretation. After adjustment for reconstructed era bands, the hospital- and ICU-mortality estimates stayed close to the primary values, and the subtype-by-late-era interaction was not significant. These findings argue against temporal drift in ICU practice or leukemia care as the explanation for the main short-term result, although the era reconstruction remained approximate. Care also changed over the 15-year study period, including BCL-2 inhibition and targeted therapies in AML and immunotherapies such as blinatumomab and CAR-T cells in ALL. These shifts may have changed subtype prognosis unevenly. A crude three-band era analysis is too coarse to capture that time-varying confounding.

The adjusted results should still be interpreted with precision in mind. The ALL subgroup contained 84 admissions and 20 hospital deaths, and confidence intervals remained wide across endpoints. In the primary hospital-mortality model, the 95% CI for ALL status ranged from 0.485 to 1.689, leaving room for a more modest subtype-specific effect. Nonetheless, the large crude ALL advantage was substantially reduced after measured baseline severity and case mix were accounted for.

The comparator group was also almost entirely AML. Although the manuscript retains the administratively accurate term non-ALL, the clinical comparison is effectively ALL versus a predominantly AML ICU cohort. The dedicated ALL-versus-AML-only sensitivity analysis yielded nearly identical ALL estimates, indicating that the seven non-AML, non-ALL admissions contributed little to the principal findings. Generalization is strongest for acute leukemia ICU cohorts and weaker for other hematologic ICU populations such as lymphoma or plasma-cell disorders, which follow different biologic and critical-care trajectories. In addition, the cohort is mixed rather than uniformly high-acuity: most admissions received no invasive organ support in the first 24 h. The results therefore apply to mixed-acuity acute-leukemia ICU cohorts and should not be assumed to extend to exclusively high-acuity populations such as those requiring mechanical ventilation or vasopressor support.

Two design features strengthen interpretation. Reporting both confounder-only and severity-adjusted estimates reduces reliance on one SOFA specification, and the decomposition analysis shows where the crude subtype contrast is absorbed. E-values, bootstrap correction, cluster-robust standard errors, and tipping-point checks add transparency, but they cannot remove the limits of these ICU-selected cohorts.

Important limitations remain. This was a retrospective database analysis (MIMIC-IV single-center; eICU multicenter), and residual confounding remains possible. Diagnosis codes defined the cohort. Remission status was available only coarsely through 6th-digit ICD specificity and ICD-9 remission-specific codes; most admissions were coded as not having achieved remission (**Supplementary Material**). Relapsed or refractory disease beyond this coarse administrative flag, transplantation history, treatment phase, and cytogenetic and molecular risk were not available. These leukemia-specific features may carry more prognostic information than subtype label, limiting biologic interpretation of the adjusted ALL estimate and making ALL-specific ICU risk subclassification inappropriate in this dataset.

The paired models share this unmeasured-confounding problem. Agreement between the confounder-only and severity-adjusted estimates addresses the SOFA-as-mediator concern only; it does not close the shared gap from unavailable leukemia-specific variables. If first-day SOFA acts as a collider rather than, or in addition to, a mediator, conditioning on it could induce bias. The severity-adjusted estimate is therefore an association, not a causal subtype effect. Some patients contributed more than one qualifying admission. Cluster-robust sensitivity analysis reduced this concern but could not remove it.

Selection bias and follow-up ascertainment also shape interpretation. The cohort was conditioned on ICU admission, and ALL-coded admissions were less likely than AML-coded admissions to enter the ICU. The marginal subtype-balance check adjusted only the overall subtype mix and did not correct ICU selection. Conditioning on ICU admission can also change the baseline characteristics under comparison; the lower first-day severity among ALL admissions may partly reflect triage patterns rather than subtype biology. The severity-adjusted estimate is therefore a within-ICU association. Longer-horizon mortality was all-cause and depended on available MIMIC-IV death ascertainment. Because individual post-discharge censoring dates were unavailable, fixed-time follow-up and Cox analyses are secondary. Finally, calendar-era reconstruction was approximate because MIMIC-IV dates are privacy-shifted, and the ALL subgroup was small, limiting interaction and subgroup analyses.

## Conclusion

Among adults with acute leukemia admitted to the ICU in MIMIC-IV, ALL admissions had lower crude hospital mortality than the predominantly AML comparator (23.8% vs. 38.6%). In the severity-adjusted primary model, the difference was not statistically significant (adjusted OR 0.905, 95% CI 0.485-1.689); the confounder-only model that omitted first-day SOFA gave a similar, also non-significant estimate (0.688, 0.385-1.228). An independent eICU cohort (n=82; 52 ALL, 30 non-ALL) showed the same crude direction: ALL was younger and had lower hospital mortality. Its severity-adjusted ALL estimate was 0.445 (95% CI 0.113-1.752), remained non-significant, and moved further from the null rather than reproducing the MIMIC-IV attenuation pattern. Baseline severity and ICD-derived remission status were the clearest measured short-term signals. These are within-ICU associations from cohorts selected by intensive care admission, with incomplete capture of leukemia-specific prognostic factors; the wide confidence intervals in both databases mean that a modest subtype effect cannot be excluded. Longer-horizon estimates remain secondary because post-discharge death ascertainment was incomplete in MIMIC-IV.

## Data Availability

Publicly available datasets were analyzed in this study. The data analyzed in this study are available from the MIMIC-IV repository on PhysioNet: https://physionet.org/content/mimiciv/. No accession number applies to this dataset.

## References

[1] Chean D Luque-Paz D Poole D Fodil S Lengline E Dupont T . Critically ill adult patients with acute leukemia: a systematic review and meta-analysis. Ann Intensive Care. (2025) 15:9. doi: 10.1186/s13613-024-01409-9 39821042 PMC11739448

[2] Park HY Suh GY Jeon K Koh WJ Chung MP Kim H . Outcome and prognostic factors of patients with acute leukemia admitted to the intensive care unit for septic shock. Leuk Lymphoma. (2008) 49:1929–34. doi: 10.1080/10428190802353609 18949617

[3] Pohlen M Thoennissen NH Braess J Thudium J Schmid C Kochanek M . Patients with acute myeloid leukemia admitted to intensive care units: outcome analysis and risk prediction. PloS One. (2016) 11:e0160871. doi: 10.1371/journal.pone.0160871 27575819 PMC5004890

[4] Kraguljac AP Croucher D Christian M Ibrahimova N Kumar V Jacob G . Outcomes and predictors of mortality for patients with acute leukemia admitted to the intensive care unit. Can Respir J. (2016) 2016:3027656. doi: 10.1155/2016/3027656 27445524 PMC4944052

[5] Halpern AB Culakova E Walter RB Lyman GH . Association of risk factors, mortality, and care costs of adults with acute myeloid leukemia with admission to the intensive care unit. JAMA Oncol. (2017) 3:374–81. doi: 10.1001/jamaoncol.2016.4858 27832254 PMC5344736

[6] Ferreyro BL Scales DC Wunsch H Cheung MC Gupta V Saskin R . Critical illness in patients with hematologic Malignancy: a population-based cohort study. Intensive Care Med. (2021) 47:1104–14. doi: 10.1007/s00134-021-06502-2 34519845

[7] van der Zee EN Termorshuizen F Benoit DD de Keizer NF Bakker J Kompanje EJO . One-year mortality of cancer patients with an unplanned ICU admission: a cohort analysis between 2008 and 2017 in the Netherlands. J Intensive Care Med. (2022) 37:1165–73. doi: 10.1177/08850666211054369 34787492 PMC9396560

[8] Chen CL Wang ST Cheng WC Wu BR Liao WC Hsu WH . Outcomes and prognostic factors in critical patients with hematologic Malignancies. J Clin Med. (2023) 12:958. doi: 10.3390/jcm12030958 36769606 PMC9918099

[9] MacPhail A Dendle C Slavin M Weinkove R Bailey M Pilcher D . Sepsis mortality among patients with haematological Malignancy admitted to intensive care 2000-2022: a binational cohort study. Crit Care. (2024) 28:148. doi: 10.1186/s13054-024-04932-0 38711155 PMC11075186

[10] Maeng CV Christiansen CF Liu KD Kamper P Christensen S Medeiros BC . Factors associated with risk and prognosis of intensive care unit admission in patients with acute leukemia: a Danish nationwide cohort study. Leuk Lymphoma. (2022) 63:2290–300. doi: 10.1080/10428194.2022.2074984 35583300

[11] Maeng CV Ostgard LSG Christiansen CF Liu KD . Changes in intensive care unit admission rates, organ support, and mortality in patients with acute myeloid leukaemia over a 12-year period: a Danish nationwide cohort study. Br J Haematol. (2021) 195:137–40. doi: 10.1111/bjh.17630 34101163 PMC8478792

[12] Vijenthira A Chiu N Jacobson D Freedman J Fralick M Tavares M . Predictors of intensive care unit admission in patients with hematologic Malignancy. Sci Rep. (2020) 10:21145. doi: 10.1038/s41598-020-78114-7 33273653 PMC7713054

[13] Slavin SD Fenech A Jankowski AL Marra A Haslam A Presley CJ . Outcomes for older adults with acute myeloid leukemia after an intensive care unit admission. Cancer. (2019) 125:3845–52. doi: 10.1002/cncr.32397 31299106 PMC6788935

[14] Saillard C Elkaim E Rey J Blaise D Vey N Faucher M . Early preemptive ICU admission for newly diagnosed high-risk acute myeloid leukemia patients. Leuk Res. (2018) 68:29–31. doi: 10.1016/j.leukres.2018.02.015 29522988

[15] Johnson AEW Bulgarelli L Shen L Gayles A Shammout A Horng S . MIMIC-IV, a freely accessible electronic health record dataset. Sci Data. (2023) 10:1. doi: 10.1038/s41597-022-01899-x 36596836 PMC9810617

[16] Vincent JL Moreno R Takala J Willatts S De Mendonca A Bruining H . The SOFA (Sepsis-related Organ Failure Assessment) score to describe organ dysfunction/failure. Intensive Care Med. (1996) 22:707–10. doi: 10.1007/BF01709751 8844239

[17] Le Gall JR Lemeshow S Saulnier F . A new Simplified Acute Physiology Score (SAPS II) based on a European/North American multicenter study. JAMA. (1993) 270:2957–63. doi: 10.1001/jama.1993.03510240069035 8254858

[18] von Elm E Altman DG Egger M Pocock SJ Gotzsche PC Vandenbroucke JP . The Strengthening the Reporting of Observational Studies in Epidemiology (STROBE) statement: guidelines for reporting observational studies. J Clin Epidemiol. (2008) 61:344–9. doi: 10.1016/j.jclinepi.2007.11.008 18313558

[19] Benchimol EI Smeeth L Guttmann A Harron K Moher D Petersen I . The REporting of studies Conducted using Observational Routinely-collected health Data (RECORD) statement. PloS Med. (2015) 12:e1001885. doi: 10.1371/journal.pmed.1001885 26440803 PMC4595218

