# Supplementary material for: Short-term mortality after intensive care admission in acute lymphoblastic versus predominantly acute myeloid leukemia: a retrospective cohort study using MIMIC-IV with eICU external replication
Source: Front Oncol. 2026 Sep 3;16:1928507. doi: 10.3389/fonc.2026.1928507 (PMC13581685; doi:10.3389/fonc.2026.1928507)
Supplement: Supplementary file 1 [file DataSheet1.docx]

**Supplementary Material**

**Supplementary File 1**

**Calendar-time (treatment-era) sensitivity analysis**

Approximate real-world calendar year was reconstructed from the MIMIC-IV privacy-preserving date-offset structure, using the midpoint of the reported anchor-year interval plus the difference between the admission year and the patient-specific anchor year. No additional integer rounding was applied before era assignment. Admissions were grouped into prespecified treatment eras (2008-2013, 2014-2017, and 2018-2022); a COVID-period sensitivity window (2020-2022) was also examined. Mapped calendar years spanned approximately 2009.0 to 2024.0; because this midpoint-based reconstruction yields approximate rather than exact calendar years, four admissions (three ALL and one non-ALL, all mapping to 2023.0-2024.0) fell slightly beyond the prespecified 2008-2022 window and were excluded from the era-banded models, leaving n = 482 complete cases.

**Era-stratified crude mortality**

| **Era** | **ALL n** | **Non-ALL n** | **ALL hospital mortality** | **Non-ALL hospital mortality** | **ALL ICU mortality** | **Non-ALL ICU mortality** |
| --- | --- | --- | --- | --- | --- | --- |
| 2008-2013 | 36 | 178 | 27.8% | 36.0% | 8.3% | 16.9% |
| 2014-2017 | 19 | 96 | 15.8% | 40.6% | 5.3% | 17.7% |
| 2018-2022 | 26 | 127 | 26.9% | 40.9% | 19.2% | 20.5% |

Crude hospital mortality was lower in ALL than in the predominantly AML comparator in every treatment era. The COVID-period (2020-2022) window contributed 84 admissions (17 ALL and 67 non-ALL).

**Era-adjusted subtype effect**

All models use the primary covariate set (ALL status, age, sex, Charlson comorbidity index, first-day SOFA) on n = 482 admissions.

| **Model** | **Outcome** | **ALL OR** | **95% CI** | **P** |
| --- | --- | --- | --- | --- |
| Severity-adjusted primary (n=482) | Hospital mortality | 0.934 | 0.499-1.749 | 0.831 |
| Era-adjusted (era indicators) | Hospital mortality | 0.932 | 0.496-1.750 | 0.826 |
| COVID-period-adjusted (2020-2022 indicator) | Hospital mortality | 0.932 | 0.498-1.747 | 0.827 |
| Severity-adjusted primary (n=482) | ICU mortality | 1.097 | 0.464-2.596 | 0.833 |
| Era-adjusted (era indicators) | ICU mortality | 1.065 | 0.447-2.538 | 0.886 |
| COVID-period-adjusted (2020-2022 indicator) | ICU mortality | 1.087 | 0.457-2.586 | 0.850 |

After addition of era indicators or a COVID-period indicator, the adjusted ALL estimate remained materially unchanged relative to the severity-adjusted primary model on the same n = 482 subset.

**Subtype-by-era interaction and COVID-period effect**

An ALL x late-era (2018-2022) interaction term was added to the era-adjusted model to test temporal effect modification. The interaction was not conventionally significant for hospital mortality (OR 1.524, 95% CI 0.404-5.751; P=0.534) or ICU mortality (OR 4.333, 95% CI 0.777-24.144; P=0.094), although the ICU-mortality interaction estimate was imprecise given the small number of ICU deaths. The COVID-period (2020-2022) indicator itself was not independently associated with hospital mortality (OR 1.108, 95% CI 0.640-1.917; P=0.715) or ICU mortality (OR 1.320, 95% CI 0.667-2.615; P=0.425). These results reduce concern that the primary short-term association is an artifact of temporal drift in leukemia treatment or ICU practice over 2008-2022.

**Supplementary File 2**

**Role of first-day SOFA: with-SOFA vs without-SOFA models**

To clarify the intended role of first-day SOFA, the short-term models were refit with and without SOFA while retaining age, sex, and Charlson comorbidity index. All models used the full n = 486 cohort.

| **Outcome** | **Model** | **n (events)** | **ALL OR** | **95% CI** | **P** |
| --- | --- | --- | --- | --- | --- |
| Hospital mortality | Without SOFA (age, sex, Charlson) | 486 (175) | 0.688 | 0.385-1.228 | 0.206 |
| Hospital mortality | With SOFA (primary model) | 486 (175) | 0.905 | 0.485-1.689 | 0.753 |
| ICU mortality | Without SOFA (age, sex, Charlson) | 486 (82) | 0.690 | 0.320-1.492 | 0.346 |
| ICU mortality | With SOFA (primary model) | 486 (82) | 1.076 | 0.455-2.540 | 0.868 |

When first-day SOFA was omitted, the adjusted ALL estimate remained below 1.0 for both hospital and ICU mortality. After SOFA was added, the estimates moved closer to the null. This attenuation is not interpreted causally. Some SOFA components may reflect downstream leukemia-related complications, such as tumor lysis affecting bilirubin or renal components and marrow failure affecting platelet-related coagulation. First-day SOFA may therefore sit partly on a subtype-to-outcome pathway. The with-SOFA estimate is a severity-adjusted estimate, whereas the without-SOFA estimate is closer to the overall subtype association. For this reason, the two estimates are reported side by side, and crude-to-adjusted attenuation is not used as a causal claim.

**Covariate-block decomposition of the attenuation**

To quantify how each covariate block contributed to attenuation of the subtype effect, sequential cumulative logistic models were fit (ALL alone; ALL plus age, sex, and Charlson comorbidity index; and the primary model additionally including first-day SOFA) on the same n = 486 complete cases. Attenuation shares are computed on the log-odds scale (movement toward the null). Because sequential attenuation depends on the order of covariate entry, each block's incremental value is also reported when that block is entered last, using likelihood-ratio tests and the change in the C-statistic (delta-C). C-statistics are Mann-Whitney AUCs of the fitted model. Validation: the with-SOFA and without-SOFA ALL estimates and the primary C-statistics (0.769 hospital, 0.813 ICU) reproduced the published values exactly.

**Table A. Sequential attenuation of the ALL effect**

| **Outcome** | **Step** | **ALL aOR (95% CI)** | **P** | **C-stat** | **delta-log-OR vs crude** | **Share of total attenuation** |
| --- | --- | --- | --- | --- | --- | --- |
| Hospital mortality | Crude (ALL only) | 0.498 (0.290-0.855) | 0.012 | 0.546 | +0.000 | - |
| Hospital mortality | + Demographics (age/sex/Charlson) | 0.688 (0.385-1.228) | 0.206 | 0.654 | +0.323 | 54% |
| Hospital mortality | + SOFA (primary model) | 0.905 (0.485-1.689) | 0.753 | 0.769 | +0.274 | 46% |
| ICU mortality | Crude (ALL only) | 0.541 (0.259-1.130) | 0.102 | 0.538 | +0.000 | - |
| ICU mortality | + Demographics (age/sex/Charlson) | 0.690 (0.320-1.492) | 0.346 | 0.611 | +0.244 | 36% |
| ICU mortality | + SOFA (primary model) | 1.076 (0.455-2.540) | 0.868 | 0.813 | +0.443 | 64% |

**Table B. Incremental value of each block (added last, likelihood-ratio test)**

| **Outcome** | **Block** | **LR χ²** | **df** | **P** | **delta-C (added last)** |
| --- | --- | --- | --- | --- | --- |
| Hospital mortality | ALL (added last) | 0.099 | 1 | 0.752 | +0.001 |
| Hospital mortality | SOFA (added last) | 68.356 | 1 | <0.001 | +0.116 |
| Hospital mortality | Demographics age/sex/Charlson (added last) | 20.895 | 3 | <0.001 | +0.025 |
| ICU mortality | ALL (added last) | 0.027 | 1 | 0.868 | +0.000 |
| ICU mortality | SOFA (added last) | 85.221 | 1 | <0.001 | +0.202 |
| ICU mortality | Demographics age/sex/Charlson (added last) | 4.707 | 3 | 0.195 | +0.011 |

**Table C. Standalone discrimination (C-statistic) of single-block models**

| **Outcome** | **Model** | **C-stat** |
| --- | --- | --- |
| Hospital mortality | ALL only | 0.546 |
| Hospital mortality | Demographics only | 0.652 |
| Hospital mortality | SOFA only | 0.742 |
| Hospital mortality | Full primary | 0.769 |
| ICU mortality | ALL only | 0.538 |
| ICU mortality | Demographics only | 0.603 |
| ICU mortality | SOFA only | 0.801 |
| ICU mortality | Full primary | 0.813 |

**Interpretation**

For hospital mortality, demographics (age, sex, Charlson comorbidity index) accounted for slightly more of the log-odds attenuation (54%) than first-day SOFA (46%); for ICU mortality, SOFA accounted for the larger share (64%) and demographics for 36%. SOFA was nonetheless the strongest single discriminator in both cases (standalone C-statistic 0.742 hospital and 0.801 ICU), and when entered last it provided the largest incremental value (delta-C +0.116 and +0.202; likelihood-ratio P<0.001 for both). ALL status, by contrast, provided essentially no incremental value when entered last (delta-C approximately 0.001; P=0.752 hospital and 0.868 ICU). Because first-day SOFA partly lies on a subtype-to-outcome pathway (see Supplementary Figure S2), the near-zero incremental value of ALL beyond SOFA is consistent with over-adjustment and does not exclude a clinically meaningful subtype effect.

**Supplementary Figure S2. Causal diagram: intended role of first-day SOFA score**

Causal diagram showing first-day SOFA lying on a subtype-driven pathway (leukemia subtype -> leukemia-related acute complications -> first-day SOFA -> short-term mortality), alongside measured baseline confounders (age, sex, Charlson comorbidity index) and other acute ICU illness. Because first-day SOFA partly mediates the subtype effect, the with-SOFA model is interpreted as severity adjustment rather than as a causal estimator of the total subtype effect.

**Supplementary File 3**

**Supplementary Material: Time-to-Event Follow-up Analyses**

These supplementary analyses were added to address the time-to-event structure of follow-up mortality after ICU admission. In addition to fixed-time 90-day and 365-day mortality analyses, we performed Kaplan-Meier estimation, log-rank testing, and Cox proportional hazards modeling for all-cause mortality through 365 days from ICU admission.

Because deaths during the index hospitalization contribute heavily to ICU-origin follow-up mortality, we also performed a hospital-survivor landmark sensitivity analysis beginning at discharge and following survivors for up to 365 days after discharge.

**Cohort Counts**

- ICU-origin 365-day survival analysis: 486 admissions, 315 deaths within 365 days.

- Post-discharge landmark survival analysis: 311 hospital survivors, 143 deaths within 365 days after discharge.

**Unadjusted Survival Comparisons**

| **Analysis** | **Log-rank chi-square** | **P value** |
| --- | --- | --- |
| ICU-origin 365-day survival | 9.83 | 0.002 |
| Post-discharge 365-day landmark survival | 3.62 | 0.057 |

**Adjusted Cox Models for ALL Status**

| **Analysis** | **N** | **Events** | **Adjusted HR for ALL** | **95% CI** | **P value** | **Concordance** | **PH test P for ALL** |
| --- | --- | --- | --- | --- | --- | --- | --- |
| ICU-origin 365-day Cox model | 486 | 315 | 1.010 | 0.718 to 1.422 | 0.953 | 0.706 | 0.846 |
| Post-discharge 365-day landmark Cox model | 311 | 143 | 0.976 | 0.606 to 1.570 | 0.919 | 0.672 | 0.213 |
| ICU-origin 365-day Cox model stratified by SOFA tertile | 486 | 315 | 0.932 | 0.663 to 1.308 | 0.683 | 0.634 |  |

**Interpretation**

The ICU-origin Kaplan-Meier comparison showed lower crude 365-day mortality in the ALL group, but this difference attenuated after multivariable Cox adjustment. Among hospital survivors, the post-discharge landmark analysis further reduced the apparent subgroup separation, supporting the interpretation that much of the crude follow-up difference reflects baseline severity and index-hospital selection rather than a stable subtype-specific survival effect.

The Schoenfeld residual test suggested possible non-proportionality for continuous SOFA in the primary ICU-origin model. A sensitivity Cox model stratified by SOFA tertile produced a similarly null ALL estimate.

**Supplementary File 4**

**Supplementary Material: Selection, Follow-up Ascertainment, and Additional Sensitivity Analyses**

These supplementary analyses were added to quantify subtype-related ICU selection, clarify follow-up ascertainment through the MIMIC-IV patient-level date-of-death field, bound uncertainty from missing post-discharge death ascertainment, evaluate within-patient correlation using subject-level cluster-robust standard errors, test SAPS II substitution for SOFA, and visualize cross-validated model calibration.

**Selection Bias Quantification**

Among all adult acute leukemia admissions, ICU admission occurred in 84/840 ALL-coded admissions (10.0%) and 402/3,067 non-ALL admissions (13.1%). The crude ICU-admission risk ratio for ALL versus non-ALL was 0.76 (Yates-corrected chi-square P=0.018).

To assess how much this differential ICU selection might influence the subtype estimate within the ICU cohort, we fit a marginal subtype-balance check using simple stabilized weights derived only from subtype-specific ICU admission probabilities estimated from the full adult ALL/AML-coded admission pool. Because these weights were based on subtype alone and did not model patient-level ICU triage determinants, they merely rebalanced the marginal subtype ratio within the ICU cohort and could not probe within-subtype ICU triage selection. They were therefore interpreted as a limited sensitivity check rather than as a correction for collider or selection bias.

| **Outcome** | **N** | **Events** | **Marginal subtype-balance check adjusted OR for ALL** | **95% CI** | **P value** |
| --- | --- | --- | --- | --- | --- |
| Hospital mortality | 480 | 174 | 0.891 | 0.513 to 1.550 | 0.684 |
| ICU mortality | 480 | 81 | 1.048 | 0.497 to 2.214 | 0.901 |
| 90-day mortality from ICU admission | 480 | 234 | 0.917 | 0.548 to 1.537 | 0.743 |
| 365-day mortality from ICU admission | 480 | 310 | 0.960 | 0.573 to 1.608 | 0.876 |

**Cluster-Robust and SAPS II Sensitivity Analyses**

| **Outcome** | **Analysis** | **N** | **Events** | **Adjusted OR for ALL** | **95% CI** | **P value** |
| --- | --- | --- | --- | --- | --- | --- |
| Hospital mortality | Severity-adjusted primary MLE | 486 | 175 | 0.905 | 0.485 to 1.689 | 0.753 |
| Hospital mortality | Severity-adjusted primary with subject-level cluster-robust SE | 486 | 175 | 0.905 | 0.517 to 1.582 | 0.725 |
| Hospital mortality | SAPS II substitution sensitivity | 486 | 175 | 0.790 | 0.425 to 1.466 | 0.455 |
| ICU mortality | Severity-adjusted primary MLE | 486 | 82 | 1.076 | 0.455 to 2.540 | 0.868 |
| ICU mortality | Severity-adjusted primary with subject-level cluster-robust SE | 486 | 82 | 1.076 | 0.477 to 2.426 | 0.861 |
| ICU mortality | SAPS II substitution sensitivity | 486 | 82 | 0.892 | 0.382 to 2.080 | 0.791 |
| 90-day mortality from ICU admission | Severity-adjusted primary MLE | 486 | 236 | 0.947 | 0.534 to 1.681 | 0.853 |
| 90-day mortality from ICU admission | Severity-adjusted primary with subject-level cluster-robust SE | 486 | 236 | 0.947 | 0.538 to 1.669 | 0.851 |
| 90-day mortality from ICU admission | SAPS II substitution sensitivity | 486 | 236 | 0.840 | 0.476 to 1.481 | 0.546 |
| 365-day mortality from ICU admission | Severity-adjusted primary MLE | 486 | 315 | 0.957 | 0.547 to 1.675 | 0.879 |
| 365-day mortality from ICU admission | Severity-adjusted primary with subject-level cluster-robust SE | 486 | 315 | 0.957 | 0.498 to 1.840 | 0.896 |
| 365-day mortality from ICU admission | SAPS II substitution sensitivity | 486 | 315 | 0.874 | 0.503 to 1.521 | 0.635 |

Follow-up Ascertainment Through the Date-of-Death Field

In the ICU follow-up cohort, a post-discharge date of death was recorded for 375/486 admissions (77.2%).

Among 311 hospital survivors, 200 (64.3%) had a recorded post-discharge death date, including 143 deaths within 365 days after discharge and 57 additional recorded deaths occurring after 365 days. The remaining 111 survivors (35.7%) had no recorded post-discharge death date and were therefore treated as alive at the fixed 365-day landmark in the main logistic analyses.

Among hospital survivors with a recorded post-discharge death, the median time from discharge to death was 170.3 days (IQR 40.3 to 439.6), with a maximum observed interval of 5315.3 days.

Because MIMIC-IV does not provide a patient-specific post-discharge censoring date other than the presence or absence of a recorded death date, a finer censoring-time distribution could not be reconstructed for survivors without a recorded death date; this limitation motivated the combined fixed-time and time-to-event presentation in the main manuscript.

Extreme-Case Bounds for Missing Post-Discharge Death Dates

To illustrate the possible impact of incomplete post-discharge death ascertainment, we calculated survivor-only extreme-case bounds for ICU-origin 365-day mortality. Among 311 hospital survivors, 140 were known to have died within 365 days of ICU admission and 111 had no recorded post-discharge death date. Under a best-case assumption that all 111 survivors without a recorded death date were alive at 365 days, survivor-only ICU-origin 365-day mortality would be 140/311 (45.0%). Under a worst-case assumption that all 111 had actually died by the same landmark, the corresponding rate would be 251/311 (80.7%). The ICU-origin anchor used here (140 deaths within 365 days of ICU admission) differs from the discharge-anchored landmark analysis in Supplementary Table 8 (143 deaths within 365 days after discharge among hospital survivors): because discharge follows ICU admission, the discharge-anchored window extends further and captures a small number of additional late deaths, so the two counts are both internally consistent within their respective landmark definitions.

Subtype-specific survivor-only bounds were similarly wide. In the ALL subgroup, ICU-origin 365-day mortality among hospital survivors ranged from 22/64 (34.4%) to 53/64 (82.8%). In the non-ALL subgroup, the corresponding range was 118/247 (47.8%) to 198/247 (80.2%). These bounds apply to the ICU-origin 365-day landmark among hospital survivors and should not be numerically equated with the separate count of 143 deaths occurring within 365 days after hospital discharge, which uses discharge rather than ICU admission as the time origin.

**Supplementary Figure S1. Cross-validated calibration of the primary models**

Ten-fold cross-validated decile-based calibration curves for the primary logistic models are shown in Supplementary Figure S1. These curves were generated from out-of-fold predictions to avoid the tautological perfect calibration seen with in-sample logistic predictions.

| **Outcome** | **N** | **Events** | **Cross-validated AUC** | **Calibration intercept** | **Calibration slope** |
| --- | --- | --- | --- | --- | --- |
| Hospital mortality | 486 | 175 | 0.763 | -0.000 | 0.951 |
| ICU mortality | 486 | 82 | 0.798 | -0.006 | 0.931 |
| 90-day mortality from ICU admission | 486 | 236 | 0.757 | -0.001 | 0.933 |
| 365-day mortality from ICU admission | 486 | 315 | 0.758 | 0.002 | 0.946 |

The Cox concordance values reported in the time-to-event supplement are Harrell C statistics and should not be interpreted as directly interchangeable with the logistic-model AUC values reported here.

**Supplementary File 5**

Supplementary: Date-of-Death Ascertainment Sensitivity and Tipping-Point Analysis

Cohort: 486 first ICU stays (ALL n=84; non-ALL n=402). primary (severity-adjusted) 365-day mortality model: mort365 ~ is_all + age + sex + Charlson + first-day SOFA.

S1. Date-of-death ascertainment structure (verified against the MIMIC-IV patient-level date-of-death field)

| **Stratum** | **N** | recorded death date present | no recorded death date |
| --- | --- | --- | --- |
| Whole cohort | 486 | 375 (77.2%) | 111 (22.8%) |
| Hospital survivors | 311 | 200 (64.3%) | 111 (35.7%) |
| Hospital deaths | 175 | 175 (100%) | 0 |

- Every in-hospital death has a recorded death time (deathtime); therefore all 111 patients without a recorded post-discharge death date are hospital survivors.

- Among the 200 hospital survivors with a recorded post-discharge death date, all 200 are deaths (143 within 365 days of discharge, 57 after 365 days). A recorded death date in a hospital survivor therefore indicates a captured post-discharge death.

- The 111 hospital survivors without a recorded post-discharge death date are the sole source of ascertainment uncertainty. Stratified by subtype: ALL 31, non-ALL 80.

- Current primary analysis treats these 111 as alive at the 365-day landmark. 365-day mortality = 315/486 (64.8%); primary-model ALL aOR = 0.957 (95% CI 0.547-1.675), P=0.879.

S2. Bounds on 365-day mortality under date-of-death ascertainment uncertainty

Only the 111 hospital survivors without a recorded post-discharge death date contribute uncertainty. Two complementary framings are reported:

(a) Survivor-only, ICU-origin 365-day mortality (denominator = 311 hospital survivors): the framing used in the main-text Results:

| Assumption for the 111 survivors without a recorded post-discharge death date | **Deaths / 311** | **Rate** |
| --- | --- | --- |
| All alive at 365 d (lower bound) | 140 / 311 | 45.0% |
| All died within 365 d (upper bound) | 251 / 311 | 80.7% |

Subtype-specific survivor-only ranges: ALL 22/64-53/64 (34.4%-82.8%); non-ALL 118/247-198/247 (47.8%-80.2%).

(b) Whole-cohort, ICU-origin 365-day mortality (denominator = 486, the primary model population):

| **Assumption for the 111** | **Deaths / 486** | **Rate** |
| --- | --- | --- |
| All alive (current primary analysis) | 315 / 486 | 64.8% |
| All died within 365 d (implausible upper bound) | 426 / 486 | 87.7% |

The two framings differ only in denominator (311 survivors vs 486 cohort) and are both assumption-free. The tipping-point analysis in S3-S5 operates on the whole-cohort primary model (486 stays) used for the adjusted subtype comparison. Upper bounds are biologically implausible but quantify the ceiling of ascertainment uncertainty.

S3. Tipping-point analysis: does the null ALL effect survive imputation?

Deaths were imputed among the 111 survivors without a recorded post-discharge death date (assigned first to the sickest within each subtype by descending first-day SOFA, then age). The primary 365-day logistic was refitted under each scenario.

| **Scenario** | **Imputed deaths (ALL / non-ALL of the 111)** | **ALL aOR (95% CI)** | **P** | **Result** |
| --- | --- | --- | --- | --- |
| S0 Current (all 111 alive) | 0 / 0 | 0.957 (0.547–1.675) | 0.879 | null |
| A Symmetric at observed death-date capture rate (~67-71%) | 21 / 57 | 1.428 (0.737–2.768) | 0.291 | null |
| B All 111 die | 31 / 80 | 1.323 (0.617–2.837) | 0.472 | null |

Both symmetric scenarios remain non-significant. Even under the pessimistic symmetric assumption that ~70% of unascertained survivors died (Scenario A), the ALL effect remained null.

**S4. Differential tipping thresholds**

To flip the conclusion to statistical significance, the imputed death pattern must be strongly differential across subtypes, i.e., one subtype's survivors without a recorded post-discharge death date assumed to have died while the other's assumed to have survived.

| **Direction of flip** | **Imputation required** | **ALL aOR (95% CI)** | **P** |
| --- | --- | --- | --- |
| Toward "ALL protective" (upper CI < 1) | 32/80 (40%) of non-ALL survivors without a recorded post-discharge death date died, and 0/31 ALL died | 0.552 (0.306–0.997) | 0.049 |
| Toward "ALL harmful" (lower CI > 1) | 11/31 (35%) of ALL survivors without a recorded post-discharge death date died, and 0/80 non-ALL died | 1.829 (1.018–3.287) | 0.043 |

**S5. Interpretation**

The null adjusted association between ALL status and 365-day mortality is robust to any symmetric imputation of the 111 hospital survivors without a recorded post-discharge death date, including the extreme case in which all 111 are assumed to have died. A statistically significant ALL effect can be recovered only under a strongly differential missing-not-at-random mechanism in which roughly 35-40% of one subtype's unascertained survivors are assumed to have died while none of the other subtype's are. Such a pattern is extreme, asymmetric between subtypes, and not supported by the observed ascertainment data. The date-of-death ascertainment limitation therefore narrows the precision of the 365-day estimate but does not threaten the qualitative null conclusion.

**Supplementary File 6**

**Supplementary Material: Exploratory Complication-Augmented Models**

These exploratory models were designed to test whether common admission-level acute complication codes added prognostic information beyond the ventilation-adjusted sensitivity model. Because sepsis, respiratory failure, and acute kidney injury were identified from admission-wide ICD coding rather than from time-anchored ICU baseline ascertainment, these analyses should be interpreted as exploratory and hypothesis-generating rather than as clean baseline prediction models.

**Summary**

| **Outcome** | **Model** | **N** | **Events** | **ALL OR** | **ALL 95% CI** | **ALL P** | **SOFA OR** | **SOFA 95% CI** | **SOFA P** | **AUC** | **Brier** | **HL P** |
| --- | --- | --- | --- | --- | --- | --- | --- | --- | --- | --- | --- | --- |
| Hospital mortality | Ventilation-adjusted sensitivity | 486 | 175 | 0.937 | 0.500-1.758 | 0.840 | 1.311 | 1.216-1.414 | <0.001 | 0.770 | 0.185 | 0.636 |
| Hospital mortality | Ventilation-adjusted sensitivity + sepsis | 486 | 175 | 0.980 | 0.513-1.872 | 0.952 | 1.266 | 1.172-1.367 | <0.001 | 0.792 | 0.177 | 0.306 |
| Hospital mortality | Ventilation-adjusted sensitivity + sepsis/respiratory failure/AKI | 486 | 175 | 1.119 | 0.576-2.175 | 0.739 | 1.248 | 1.152-1.352 | <0.001 | 0.810 | 0.170 | 0.016 |
| ICU mortality | Ventilation-adjusted sensitivity | 486 | 82 | 0.994 | 0.418-2.363 | 0.990 | 1.298 | 1.190-1.417 | <0.001 | 0.824 | 0.108 | 0.766 |
| ICU mortality | Ventilation-adjusted sensitivity + sepsis | 486 | 82 | 0.977 | 0.407-2.348 | 0.959 | 1.271 | 1.162-1.390 | <0.001 | 0.832 | 0.107 | 0.519 |
| ICU mortality | Ventilation-adjusted sensitivity + sepsis/respiratory failure/AKI | 486 | 82 | 0.990 | 0.406-2.410 | 0.982 | 1.270 | 1.156-1.395 | <0.001 | 0.841 | 0.108 | 0.263 |

**Likelihood-Ratio Comparison Versus the Primary Model**

| **Outcome** | **Comparison** | **LR chi-square** | **df** | **P value** |
| --- | --- | --- | --- | --- |
| Hospital mortality | Ventilation-adjusted sensitivity vs + sepsis | 19.72 | 1 | <0.001 |
| Hospital mortality | Ventilation-adjusted sensitivity vs + sepsis/respiratory failure/AKI | 35.56 | 3 | <0.001 |
| ICU mortality | Ventilation-adjusted sensitivity vs + sepsis | 3.92 | 1 | 0.048 |
| ICU mortality | Ventilation-adjusted sensitivity vs + sepsis/respiratory failure/AKI | 6.61 | 3 | 0.085 |

**Interpretation**

For hospital mortality, adding admission-level sepsis and broader complication coding improved discrimination beyond the ventilation-adjusted sensitivity model and attenuated the SOFA estimate, whereas the adjusted ALL estimate remained close to null throughout. For ICU mortality, the gain was smaller and less consistent. These findings support the descriptive impression that acute complication burden contains clinically relevant information beyond generic physiology, but the time-order ambiguity of admission-level ICD coding prevents a causal or baseline-predictive interpretation.

**Supplementary File 7**

**Supplementary Table: ICU Case Mix and Acute Complication Burden (N = 486 first ICU stays)**

This table characterizes the case mix of the analytic cohort. Acute complications were ascertained from all ICD-9/ICD-10 diagnosis codes recorded during the qualifying hospital admission (admission-level presence, not necessarily the admitting diagnosis). Calendar-era mapping used the MIMIC-IV privacy-shifted date-offset structure.

**Panel A. Early organ support and stay characteristics**

| **Characteristic** | **Value** |
| --- | --- |
| Invasive ventilation within 24 h, n (%) | 103 (21.2) |
| Any vasoactive agent within 24 h, n (%) | 100 (20.6) |
| Dialysis / RRT within 24 h, n (%) | 29 (6.0) |
| None of the above in first 24 h, n (%) | 324 (66.7) |
| ICU length of stay, days - median (IQR) | 1.91 (1.05–4.00) |
| < 1 day, % | 23.7 |
| 1–3 days, % | 43.9 |
| > 7 days, % | 14.2 |
| First-day SOFA, median (IQR) | 6.0 (4.0–8.0) |
| First-day SOFA ≥ 6, % | 52.1 |

**Panel B. Acute complications during the qualifying admission (admission-level, ICD-coded)**

| **Complication** | **n (%)** |
| --- | --- |
| Acute kidney injury | 248 (51.0) |
| Coagulopathy / bleeding / anemia | 239 (49.2) |
| Respiratory failure | 238 (49.0) |
| Sepsis / septic shock | 202 (41.6) |
| Neutropenia | 193 (39.7) |
| Pneumonia | 181 (37.2) |
| Tumor lysis syndrome | 30 (6.2) |

ICD-9 prefixes: sepsis 038/9959/78552; respiratory failure 5188/5180/7860; neutropenia 2880; AKI 584; coagulopathy/bleeding 2866/2867/2873/2874/2875/2869; pneumonia 480-487; TLS 27788.

ICD-10 prefixes: sepsis A40/A41/R652/R57; respiratory failure J80/J95/J96; neutropenia D70; AKI N17; coagulopathy/bleeding/anemia D65/D68/D69/D649; pneumonia J12-J18; TLS E883.

**Panel C. Most common primary (seq = 1) discharge diagnoses**

| **ICD code (version)** | **Diagnosis** | **n** |
| --- | --- | --- |
| 20500 (9) | Acute myeloid leukemia, without mention of having achieved remission | 85 |
| C9200 (10) | Acute myeloblastic leukemia, not having achieved remission | 67 |
| 0389 (9) | Unspecified septicemia | 17 |
| A419 (10) | Sepsis, unspecified organism | 15 |
| 20400 (9) | Acute lymphoid leukemia, without mention of having achieved remission | 14 |
| V5811 (9) / Z5111 (10) | Encounter for antineoplastic chemotherapy | 24 (combined) |
| C9100 (10) | Acute lymphoblastic leukemia, not having achieved remission | 11 |

Note: the leukemia itself or a chemotherapy encounter was the primary (seq = 1) diagnosis for the majority of admissions; acute complications were most often recorded as secondary diagnoses. The high coded complication burden (Panel B) coexists with a low rate of first-24 h invasive organ support (Panel A), reflecting medical (non-invasive) management of many acute complications and a wide acuity spectrum within the cohort.

**Panel D. ICU admission rate among adult acute leukemia admissions, by subtype (selection-bias assessment)**

| **Subgroup** | **Adult leukemia admissions** | **Admissions with ≥ 1 ICU stay** | **ICU admission rate** |
| --- | --- | --- | --- |
| ALL | 840 | 84 | 10.0% |
| Non-ALL | 3,067 | 402 | 13.1% |
| AML only (subset of non-ALL) | 3,017 | 396 | 13.1% |
| Note: Non-ALL comprises AML plus monocytic and other acute leukemia codes (402 ICU admissions = 395 AML + 6 AML-monocytic-only + 1 co-coded). Rate ratio: ALL vs non-ALL = 0.76 (P=0.018); ALL vs AML only = 0.762 (95% CI 0.610-0.952). Both comparators yield an ICU admission rate of 13.1%; the non-ALL comparator is used in the main text. |  |  |  |

ALL versus non-ALL ICU admission rate: rate ratio 0.76; Yates-corrected chi-square P=0.018. ALL-coded admissions were less likely to be admitted to ICU than the non-ALL comparator; because the analytic cohort is conditioned on ICU admission, this differential selection is a potential source of selection bias for the subtype comparison and is acknowledged as a limitation.

**Supplementary File 8**

**Supplementary Table: Variable Missingness**

| **Variable** | **Available_n** | **Missing_n** | **Missing_pct** |
| --- | --- | --- | --- |
| ld_ldh_max | 347 | 139 | 28.6 |
| first_day_urineoutput | 463 | 23 | 4.7 |
| marital_status | 473 | 13 | 2.7 |
| wbc_max | 477 | 9 | 1.9 |
| bun_max | 479 | 7 | 1.4 |
| creatinine_max | 480 | 6 | 1.2 |
| first_day_gcs_min | 481 | 5 | 1.0 |
| hemoglobin_min | 481 | 5 | 1.0 |
| platelets_min | 481 | 5 | 1.0 |
| icu_los_days | 485 | 1 | 0.2 |
| insurance | 485 | 1 | 0.2 |
| admission_location | 486 | 0 | 0.0 |
| admission_type | 486 | 0 | 0.0 |
| age | 486 | 0 | 0.0 |
| any_vasoactive_first24h | 486 | 0 | 0.0 |
| charlson_comorbidity_index | 486 | 0 | 0.0 |
| dialysis_present_first24h | 486 | 0 | 0.0 |
| first_careunit | 486 | 0 | 0.0 |
| first_day_sofa | 486 | 0 | 0.0 |
| gender | 486 | 0 | 0.0 |
| hosp_los_days | 486 | 0 | 0.0 |
| hospital_expire_flag | 486 | 0 | 0.0 |
| icu_expire_flag | 486 | 0 | 0.0 |
| invasive_vent_first24h | 486 | 0 | 0.0 |
| race | 486 | 0 | 0.0 |
| sapsii | 486 | 0 | 0.0 |

**Supplementary File 9**

**Supplementary Table: Model Comparison**

| **Outcome** | **Model** | **N** | **Events** | **C_statistic** | **Adjusted_OR_for_ALL** | **CI95_low** | **CI95_high** | **P_value** | **Covariates** |
| --- | --- | --- | --- | --- | --- | --- | --- | --- | --- |
| Hospital mortality | Unadjusted | 486 | 175 |  | 0.498 | 0.290 | 0.855 | 0.012 | None |
| ICU mortality | Unadjusted | 486 | 82 |  | 0.541 | 0.259 | 1.130 | 0.110 | None |
| Hospital mortality | Severity-adjusted primary | 486 | 175 | 0.769 | 0.905 | 0.485 | 1.689 | 0.753 | ALL status, age, sex, Charlson comorbidity index, first-day SOFA |
| ICU mortality | Severity-adjusted primary | 486 | 82 | 0.813 | 1.076 | 0.455 | 2.540 | 0.868 | ALL status, age, sex, Charlson comorbidity index, first-day SOFA |
| Hospital mortality | Ventilation-adjusted sensitivity | 486 | 175 | 0.770 | 0.937 | 0.500 | 1.758 | 0.840 | ALL status, age, sex, Charlson comorbidity index, first-day SOFA, invasive ventilation within 24 h |
| ICU mortality | Ventilation-adjusted sensitivity | 486 | 82 | 0.824 | 0.994 | 0.418 | 2.363 | 0.990 | ALL status, age, sex, Charlson comorbidity index, first-day SOFA, invasive ventilation within 24 h |
| Hospital mortality | Extended hematologic | 476 | 170 | 0.786 | 1.112 | 0.572 | 2.163 | 0.755 | Ventilation-adjusted sensitivity covariates plus WBC maximum and platelet minimum |
| ICU mortality | Extended hematologic | 476 | 81 | 0.820 | 0.978 | 0.393 | 2.437 | 0.963 | Ventilation-adjusted sensitivity covariates plus WBC maximum and platelet minimum |
| Hospital mortality | Sensitivity vasoactive | 486 | 175 | 0.772 | 0.942 | 0.501 | 1.771 | 0.853 | Ventilation-adjusted sensitivity covariates plus vasoactive-agent exposure |
| ICU mortality | Sensitivity vasoactive | 486 | 82 | 0.827 | 0.990 | 0.411 | 2.383 | 0.982 | Ventilation-adjusted sensitivity covariates plus vasoactive-agent exposure |

**Supplementary File 10**

**Supplementary Table: Unadjusted vs Adjusted Association of ALL Status With Mortality**

| **Outcome** | **Model** | **Adjusted_OR_for_ALL** | **CI95_low** | **CI95_high** | **P_value** |
| --- | --- | --- | --- | --- | --- |
| Hospital mortality | Unadjusted | 0.498 | 0.290 | 0.855 | 0.012 |
| ICU mortality | Unadjusted | 0.541 | 0.259 | 1.130 | 0.110 |
| Hospital mortality | Severity-adjusted primary | 0.905 | 0.485 | 1.689 | 0.753 |
| ICU mortality | Severity-adjusted primary | 1.076 | 0.455 | 2.540 | 0.868 |
| Hospital mortality | Ventilation-adjusted sensitivity | 0.937 | 0.500 | 1.758 | 0.840 |
| ICU mortality | Ventilation-adjusted sensitivity | 0.994 | 0.418 | 2.363 | 0.990 |
| Hospital mortality | Extended hematologic | 1.112 | 0.572 | 2.163 | 0.755 |
| ICU mortality | Extended hematologic | 0.978 | 0.393 | 2.437 | 0.963 |
| Hospital mortality | Sensitivity vasoactive | 0.942 | 0.501 | 1.771 | 0.853 |
| ICU mortality | Sensitivity vasoactive | 0.990 | 0.411 | 2.383 | 0.982 |

**Supplementary File 11**

**Supplementary Figure S3. Kaplan-Meier survival by leukemia subtype**

Kaplan-Meier curves for all-cause survival through 365 days from ICU admission by leukemia subgroup, with numbers at risk shown below the x-axis. The unadjusted log-rank comparison was significant (P=0.002), but the baseline-adjusted Cox model showed no independent association for ALL (HR 1.010, 95% CI 0.718-1.422; P=0.953), and a SOFA-tertile-stratified sensitivity Cox model was likewise null (HR 0.932, 95% CI 0.663-1.308; P=0.683), indicating that the crude curve separation narrowed after baseline severity adjustment. These supportive time-to-event analyses are limited by incomplete post-discharge death ascertainment (the MIMIC-IV date-of-death field was available for 77.2% of the ICU cohort and 64.3% of hospital survivors) and are therefore presented in the Supplementary Material rather than as main-text figures.

**Supplementary File 12**

**E-value sensitivity analysis for unmeasured confounding**

To gauge how sensitive the primary subtype-mortality estimate is to unmeasured confounding, we computed E-values, which express the minimum strength of an unmeasured confounder linking subtype to mortality that would be needed to move the estimate to the null. For common outcomes, E-values were computed from the adjusted odds ratios using the standard approximation implemented in the EValue package for evalues.OR(..., rare = FALSE), and the confidence-interval E-value was reported for the bound closest to the null.

| **Outcome** | **Primary aOR (95% CI)** | **E-value (point)** | **E-value (closest CI bound)** |
| --- | --- | --- | --- |
| Hospital mortality | 0.905 (0.485-1.689) | 1.28 | 1.00 (CI includes the null) |
| ICU mortality | 1.076 (0.455-2.540) | 1.23 | 1.00 (CI includes the null) |

The point-estimate E-values were 1.28 for hospital mortality and 1.23 for ICU mortality, so modest unmeasured confounding could move the point estimates across the null. Because both confidence intervals already crossed the null, the corresponding confidence-interval E-values were 1.00 for both outcomes. These values match the wide confidence intervals and the limits of an ICU-selected single-center cohort. The null result should therefore be treated as inconclusive rather than as evidence of no subtype effect. E-values address unmeasured confounding; they do not formally bound the collider or selection bias introduced by conditioning on ICU admission.

Supplementary File 13

ICD code definitions for acute complication variables

Acute complication variables were identified from all ICD-9 and ICD-10 diagnosis codes recorded during the qualifying hospital admission. The first three variables were also used in the exploratory complication-augmented mortality models; the remaining variables were used for descriptive case-mix reporting only.

| Complication variable | ICD-9 code prefixes / codes | ICD-10 code prefixes / codes | Use in manuscript |
| --- | --- | --- | --- |
| Sepsis / septic shock | 038, 9959, 78552 | A40, A41, R652, R57 | Descriptive case-mix and complication-augmented models |
| Respiratory failure | 5188, 5180, 7860 | J80, J95, J96 | Descriptive case-mix and complication-augmented models |
| Acute kidney injury | 584 | N17 | Descriptive case-mix and complication-augmented models |
| Pneumonia | 480-487 | J12-J18 | Descriptive case-mix only |
| Coagulopathy / bleeding / anemia | 2866, 2867, 2873, 2874, 2875, 2869 | D65, D68, D69, D649 | Descriptive case-mix only; D649 captured unspecified anemia and was not used in mortality models |
| Neutropenia | 2880 | D70 | Descriptive case-mix only |
| Tumor lysis syndrome | 27788 | E883 | Descriptive case-mix only |

Source notes: sepsis/septic shock, respiratory failure, and acute kidney injury were defined in export_complication_augmented_baseline.py, which generated the complication-augmented analysis dataset. The broader descriptive prefixes were recorded in the case-mix table source and used to summarize admission-level complication burden. D649 was retained only in the descriptive case-mix flag to capture unspecified anemia as part of hematologic complication burden; it was not used in the complication-augmented mortality models.

**Supplementary File 14. Remission status extracted from ICD code specificity**

The 6th character of the ICD-10 acute leukemia codes and the 5th digit of the ICD-9 acute leukemia codes encode remission status, so a coarse remission variable was derived for each admission from its qualifying leukemia diagnosis codes (ICD-9 204-207 series, 5th digit 0 = without remission, 1 = in remission, 2 = in relapse; ICD-10 C91.0 / C92.0 / C93.0 series, 6th character 0 = not having achieved remission, 1 = in remission, 2 = in relapse). An admission was classified as active disease if any leukemia code indicated not-in-remission or relapse, and as in-remission only if a remission code was present without any active code. Remission status was then added to the severity-adjusted primary model (remission sensitivity model), and the primary model was re-fit on the active-disease subset. A three-level classification (not-in-remission, relapse, in-remission) is also reported for descriptive purposes. These are sensitivity checks: remission is captured coarsely from administrative coding and does not reflect treatment response, measurable residual disease, or cytogenetic/molecular risk.

***Table R1. Remission status distribution and crude mortality, by ALL status.***

| Remission status | ALL n | non-ALL n | Overall n (%) | Hospital mortality n (%) | ICU mortality n (%) |
| --- | --- | --- | --- | --- | --- |
| In remission | 19 | 65 | 84 (17.3%) | 10 (11.9%) | 7 (8.3%) |
| Not in remission | 51 | 269 | 320 (65.8%) | 131 (40.9%) | 64 (20.0%) |
| Relapse | 14 | 68 | 82 (16.9%) | 34 (41.5%) | 11 (13.4%) |
| Active disease (not-in-remission or relapse) | 65 | 337 | 402 (82.7%) | 165 (41.0%) | 75 (18.7%) |
| Total | 84 | 402 | 486 (100%) | 175 (36.0%) | 82 (16.9%) |

***Table R2. Severity-adjusted primary model and remission sensitivity models for ALL status.***

| Outcome | Model | N (events) | ALL aOR (95% CI) | ALL P | Remission aOR (active vs in-remission) | C-statistic (delta-C) |
| --- | --- | --- | --- | --- | --- | --- |
| Hospital | Primary (reference) | 486 (175) | 0.905 (0.485-1.689) | 0.753 | - | 0.769 (ref) |
| Hospital | Primary + remission | 486 (175) | 0.839 (0.444-1.589) | 0.591 | 4.245 (1.936-9.311), P<0.001 | 0.786 (+0.017) |
| Hospital | Primary, active-disease subset | 402 (165) | 0.758 (0.388-1.480) | 0.416 | - | - |
| ICU | Primary (reference) | 486 (82) | 1.076 (0.455-2.540) | 0.868 | - | 0.813 (ref) |
| ICU | Primary + remission | 486 (82) | 1.038 (0.437-2.466) | 0.933 | 1.714 (0.651-4.516), P=0.275 | 0.816 (+0.003) |
| ICU | Primary, active-disease subset | 402 (75) | 1.165 (0.480-2.828) | 0.735 | - | - |

Likelihood-ratio test for adding remission to the primary model: hospital mortality chi-square 15.77, df=1, P=7.0x10-5; ICU mortality chi-square 1.27, df=1, P=0.259. Three-level classification (in-remission as reference) for hospital mortality: not-in-remission adjusted OR 4.15 (P<0.001), relapse adjusted OR 4.59 (P=0.001).

Interpretation. Remission status was strongly associated with hospital mortality: crude hospital mortality was 11.9% among in-remission admissions versus 41.0% among active-disease admissions, and active disease carried an adjusted odds ratio of 4.25 (95% CI 1.94-9.31; P<0.001) for hospital mortality, with a significant improvement in model fit (likelihood-ratio P=7.0x10-5) and discrimination (C-statistic increase 0.017). Remission was not independently associated with ICU mortality (adjusted OR 1.71, P=0.28), consistent with ICU death being driven by acute physiology rather than disease status. ALL-coded admissions had a higher in-remission rate than non-ALL admissions (22.6% vs 16.2%, P=0.16), which contributes to the lower crude mortality in ALL and is part of the case-mix difference the adjusted analysis addresses. Adjusting for remission did not materially change the ALL estimate (hospital mortality 0.905 to 0.839; ICU 1.076 to 1.038), and restricting to active-disease admissions gave a consistent non-significant ALL estimate (hospital 0.758; ICU 1.165). The primary short-term conclusion is therefore robust to remission status. Even when captured coarsely from administrative coding, remission status carried substantially more short-term prognostic information than leukemia subtype label.

**Supplementary File 15. Missing-data mechanism and multiple-imputation sensitivity**

The primary model covariates (ALL status, age, sex, Charlson comorbidity index, first-day SOFA) were complete for all 486 admissions, so the primary analysis required no imputation. Only the extended hematologic sensitivity model was affected by missing data: 9 admissions were missing white-cell count maximum and 5 were missing platelet count minimum, leaving 476/486 (98.0%) complete cases (10 dropped, 2.1%). To assess the missingness mechanism and the robustness of the complete-case approach, we (i) compared complete and incomplete cases on outcome and key covariates, and (ii) re-fit the extended hematologic model under multiple imputation. Multiple imputation used an iterative chained-equations imputer (BayesianRidge, posterior sampling, 25 iterations) with m=20 imputed datasets; the ALL log-odds and its standard error were pooled using Rubin’s rules.

***Table M1. Complete-case versus incomplete-case comparison (extended hematologic covariate set).***

| Variable | Complete cases (n=476) | Incomplete cases (n=10) | P value |
| --- | --- | --- | --- |
| Hospital mortality | 169 (35.7%) | 5 (50.0%) | 0.352 |
| ALL status | 80 (17.0%) | 3 (30.0%) | 0.283 |
| Age, median (IQR) | 63.0 (52.0, 72.0) | 65.0 | 0.992 |
| First-day SOFA, median (IQR) | 6.0 (4.0, 8.0) | 4.5 | 0.141 |
| Charlson index, median (IQR) | 6.0 (4.0, 7.0) | 6.0 | 0.648 |

P values from Pearson chi-square (binary) or Mann-Whitney U (continuous). No comparison reached significance, indicating that incomplete cases did not differ materially from complete cases and supporting a missing-at-random assumption.

Multiple-imputation result (m=20): the pooled ALL adjusted odds ratio for hospital mortality was 1.091 (95% CI 0.569-2.093), essentially identical to the complete-case estimate of 1.112 (95% CI 0.572-2.163); the difference in point estimates was 0.020 and the fraction of missing information for the ALL coefficient was 0.1%. The primary short-term conclusion is therefore robust to the missing-data handling.

**Supplementary File 16. External replication in the eICU collaborative research database**

To assess whether the short-term pattern observed in MIMIC-IV generalized to an independent cohort, we repeated the ALL-versus-predominantly-AML comparison in the eICU Collaborative Research Database (eICU-CRD), a large multicenter US ICU dataset distinct from MIMIC-IV. This analysis is interpretive and hypothesis-supporting rather than a powered confirmatory test: the available eICU acute-leukemia ICU cohort is small, and the confidence intervals are correspondingly wide.

Cohort identification. Adults (18 years or older) with a first ICU stay and acute leukemia were identified from free-text fields using the same logic applied in our prior eICU work: the diagnosis, admission diagnosis, and APACHE admission-diagnosis tables were searched for acute-leukemia terms ('acute lymphocytic', 'acute lymphoblastic', 'acute myelocytic', 'acute myeloid', 'acute leukemia', and the corresponding admission-diagnosis paths). Leukemia subtype was then assigned from the same free-text fields: admissions whose text contained 'lymphocytic' or 'lymphoblastic' were classified as ALL, and those containing 'myelocytic' or 'myeloid' as AML. eICU diagnosis coding is text-based and sparser than the ICD-9/10 coding used for MIMIC-IV, so this identification is approximate.

Outcomes and severity. Outcomes were hospital mortality (hospitaldischargestatus = 'Expired') and unit (ICU) mortality (unitdischargestatus = 'Expired'); both were complete for all 82 stays, with no post-discharge date-of-death gap. eICU encodes physiological severity as the APACHE score rather than SOFA, and it has no Charlson comorbidity index. The severity-adjusted model therefore substituted the APACHE score for first-day SOFA, and the confounder-only model used age and sex only (without Charlson). These specifications differ from the MIMIC-IV primary models and are not directly interchangeable with them.

The final eICU cohort comprised 82 first ICU stays: 52 ALL and 30 non-ALL, of whom 25 were AML (the remaining non-ALL admissions carried non-specific acute-leukemia coding without an explicit AML label). Descriptive characteristics are shown in Table E1.

*Table E1. Baseline characteristics and crude mortality of the eICU external-replication cohort.*

| **Variable** | **ALL (n=52)** | **Non-ALL (n=30)** | **AML-only (n=25)** |
| --- | --- | --- | --- |
| Age, years, median (IQR) | 58 (37-68) | 63 (44-76) | 63 (44-77) |
| Male sex, n (%) | 31 (60%) | 14 (47%) | 10 (40%) |
| APACHE score, median (IQR) | 66 | 69 | 69 |
| Hospital mortality, n (%) | 13 (25.0%) | 10 (33.3%) | 8 (32.0%) |
| Unit (ICU) mortality, n (%) | 7 (13.5%) | 4 (13.3%) | 4 (16.0%) |

As in MIMIC-IV, ALL admissions were younger than the predominantly AML comparator and had lower crude hospital mortality. Absolute APACHE values are not comparable to MIMIC-IV SOFA values.

*Table E2. ALL-versus-predominantly-AML association with short-term mortality in the eICU cohort.*

| **Comparator / model** | **Outcome** | **N (events)** | **Adjusted OR for ALL** | **95% CI** | **P** |
| --- | --- | --- | --- | --- | --- |
| ALL vs non-ALL, crude | Hospital mortality | 82 (23) | 0.667 | 0.249-1.785 | 0.420 |
| ALL vs non-ALL, confounder-only (age, sex) | Hospital mortality | 82 (23) | 0.675 | 0.234-1.949 | 0.468 |
| ALL vs non-ALL, severity-adjusted (+APACHE) | Hospital mortality | 70 (20) | 0.445 | 0.113-1.752 | 0.247 |
| ALL vs non-ALL, crude | Unit mortality | 82 (11) | 1.011 | 0.270-3.785 | 0.987 |
| ALL vs non-ALL, confounder-only (age, sex) | Unit mortality | 82 (11) | 0.922 | 0.237-3.589 | 0.906 |
| ALL vs non-ALL, severity-adjusted (+APACHE) | Unit mortality | 70 (11) | 0.388 | 0.072-2.099 | 0.272 |
| ALL vs AML-only, crude | Hospital mortality | 77 (21) | 0.708 | 0.248-2.022 | 0.519 |
| ALL vs AML-only, severity-adjusted (+APACHE) | Hospital mortality | 67 (19) | 0.470 | 0.113-1.960 | 0.300 |
| ALL vs AML-only, severity-adjusted (+APACHE) | Unit mortality | 67 (11) | 0.354 | 0.065-1.922 | 0.229 |

The severity-adjusted models included ALL status, age, sex, and APACHE score; the confounder-only model omitted APACHE. N (events) reflect complete-case analysis for the covariates in each model; the severity-adjusted models drop to n=70 (or 67 for the AML-only subcomparison) because APACHE score was available for 70 of 82 stays. P values are two-sided Wald tests and are not adjusted for multiplicity.

**Interpretation.**

At the crude level, the eICU cohort reproduced the qualitative pattern seen in MIMIC-IV: ALL admissions were younger and had lower hospital mortality. Its adjusted ALL estimate remained on the protective side of the null, but moved further from the null rather than reproducing the MIMIC-IV attenuation pattern. However, every confidence interval was wide and crossed the null, and the cohort is small (52 ALL, 20-23 hospital events), so the eICU analysis cannot independently confirm or exclude a subtype effect. Two points warrant emphasis. First, the MIMIC-IV severity-adjusted estimate (0.905) attenuated toward the null, whereas the eICU severity-adjusted estimate (0.445) was numerically more protective; the two point estimates therefore differ in magnitude. Because the 95% confidence intervals in the two cohorts overlap broadly (MIMIC-IV 0.485-1.689; eICU 0.113-1.752), because both contain the same range of clinically plausible values, and because the eICU model used APACHE in place of SOFA and omitted Charlson comorbidity, this numerical divergence should not be read as disagreement between the two databases. Second, eICU hospital-discharge mortality was complete for all stays, so this comparison is free of the post-discharge date-of-death ascertainment gap that limits the MIMIC-IV longer-horizon analyses. Taken together, the two cohorts support a single, honest inference: a crude ALL survival advantage at ICU presentation that does not reach statistical significance after adjustment, with confidence intervals too wide to confirm or rule out a modest subtype-specific effect.
